# Correlating Conformational Equilibria with Catalysis in the Electron Bifurcating EtfABCX of *Thermotoga maritima*

**DOI:** 10.1021/acs.biochem.3c00472

**Published:** 2023-11-28

**Authors:** Daniel T. Murray, Xiaoxuan Ge, Gerrit J. Schut, Daniel J. Rosenberg, Michal Hammel, Jan C. Bierma, Russ Hille, Michael W. W. Adams, Greg L. Hura

**Affiliations:** † Molecular Biophysics and Integrated Bioimaging Division, Lawrence Berkeley National Laboratory, Berkeley, California 94720, United States; ‡ Department of Biochemistry and Molecular Biology, University of Georgia, Athens, Georgia 30602, United States; § Linac Coherent Light Source, SLAC National Accelerator Laboratory, Menlo Park, California 94025, United States; ∥ Department of Biochemistry, University of California, Riverside, Riverside, California 92521, United States; ⊥ Chemistry and Biochemistry Department, University of California, Santa Cruz, Santa Cruz, California 95064, United States

## Abstract

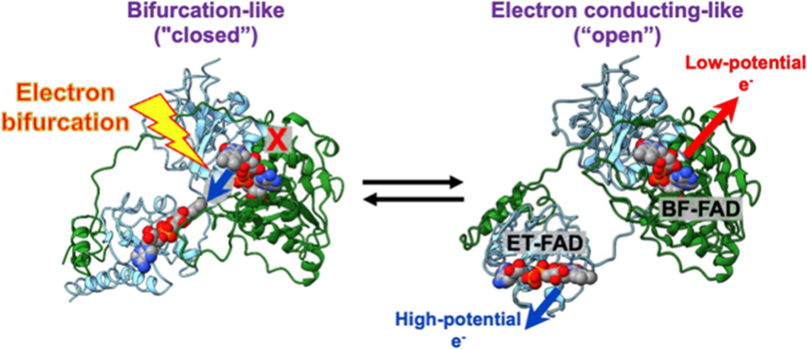

Electron bifurcation (BF) is an evolutionarily ancient energy coupling mechanism in anaerobes, whose associated enzymatic machinery remains enigmatic. In BF-flavoenzymes, a chemically high-potential electron forms in a thermodynamically favorable fashion by simultaneously dropping the potential of a second electron before its donation to physiological acceptors. The cryo-EM and spectroscopic analyses of the BF-enzyme Fix/EtfABCX from *Thermotoga maritima* suggest that the BF-site contains a special flavin-adenine dinucleotide and, upon its reduction with NADH, a low-potential electron transfers to ferredoxin and a high-potential electron reduces menaquinone. The transfer of energy from high-energy intermediates must be carefully orchestrated conformationally to avoid equilibration. Herein, anaerobic size exclusion-coupled small-angle X-ray scattering (SEC-SAXS) shows that the Fix/EtfAB heterodimer subcomplex, which houses BF- and electron transfer (ET)-flavins, exists in a conformational equilibrium of compacted and extended states between flavin-binding domains, the abundance of which is impacted by reduction and NAD(H) binding. The conformations identify dynamics associated with the *T. maritima* enzyme and also recapitulate states identified in static structures of homologous BF-flavoenzymes. Reduction of Fix/EtfABCX’s flavins alone is insufficient to elicit domain movements conducive to ET but requires a structural “trigger” induced by NAD(H) binding. Models show that Fix/EtfABCX’s superdimer exists in a combination of states with respect to its BF-subcomplexes, suggesting a cooperative mechanism between supermonomers for optimizing catalysis. The correlation of conformational states with pathway steps suggests a structural means with which Fix/EtfABCX may progress through its catalytic cycle. Collectively, these observations provide a structural framework for tracing Fix/EtfABCX’s catalysis.

Biological electron bifurcation (BF) by enzymatic machinery is an evolutionarily conserved, albeit recently investigated, form of biological energy conservation.^1−3^ BF is the process in which one electron of a donor pair is sent to a higher potential acceptor and the other to a lower potential acceptor in a thermodynamically favored manner. Enzymatic gene products that facilitate BF have been discovered in many anaerobic and aerobic microbes.^2^ The delivery of electrons to two different energy levels within the discovered BF-enzymes is dependent on flavin-adenine dinucleotide (FAD) or flavin mononucleotide (FMN) cofactors. Proteins that orchestrate flavin-based BF are now appreciated as crucial components in redox metabolism pathways of many organisms that enable normally unfavorable electron transfer (ET) reactions. Thus far, BF enzymes have been split into four phylogenetically unrelated, albeit independently evolved groups. These groups include [FeFe] hydrogenases containing HydABC, heterodisulfide reductases containing HdrA, transhydrogenases containing NfNAB, and electron transfer flavoproteins (ETFs) containing EtfAB.^4−6^ The HydABC-type hydrogenases were recently shown to be representative of the diverse and ubiquitous so-called Bfu family with a noncanonical FMN/FeS cluster-based BF mechanism.^7^ Some BF-enzymes can also catalyze the reverse of electron bifurcation, or confurcation, such as the *Acetomicrobium mobile* NiFe-HydABCSL hydrogenase that reduces NAD^+^ and ferredoxin using electrons donated from H_2_ as well as the reverse reaction in which NADH and reduced ferredoxin provide electrons for proton reduction.^8^

The availability of high-resolution structures for a handful of EtfAB systems has been essential for deciphering BF-enzyme assembly, the spatial relationship between substrates and cofactors, and catalytic mechanism. Structures are available for butyryl-CoA (Coenzyme A) dehydrogenase (EtfAB-Bcd) from *Acidaminococcus fermentans*, caffeyl-CoA reductase (CarCDE) from *Acetobacterium woodii*, and Fix/EtfABCX from *Thermotoga maritima*.^9−11^ EtfAB-Bcd and CarCDE both reduce high-potential CoA-derivatives (high-potential acceptors) along with the low-potential acceptor ferredoxin (Fd) via NADH oxidation and possess structures obtained through X-ray crystallography (MX; PDB IDs: 4L2I and 6FAH, respectively), whereas Fix/EtfABCX reduces the high-potential acceptor menaquinone (MQ) along with Fd and whose structure was solved by cryoelectron microscopy (cryo-EM; PDB ID: 7KOE). *A. woodii* CarCDE and *Clostridium difficile* EtfAB-Bcd are both soluble heterododecameric “supertetramers” (Car(CDE)_4_ and (EtfAB(Bcd))_4_, respectively).^12^ Alternatively, *A. fermentans* EtfAB-Bcd is a dissociable complex between two EtfAB heterodimers and a Bcd tetramer while *T. maritima* Fix/EtfABCX is a membrane-associated “superdimer” of EtfABCX protomers (Fix/Etf(ABCX)_2_; “EtfABCX” for simplicity hereafter; Figure 1A), with two copies of an ABCX heterotetramer protomer or “supermonomer” that share a dimerization interface at EtfC. The EtfCs of the superdimer complex are in turn membrane-associated. Each supermonomer is in possession of an independent BF module in the form of discrete EtfAB subunits.

**Figure 1:**
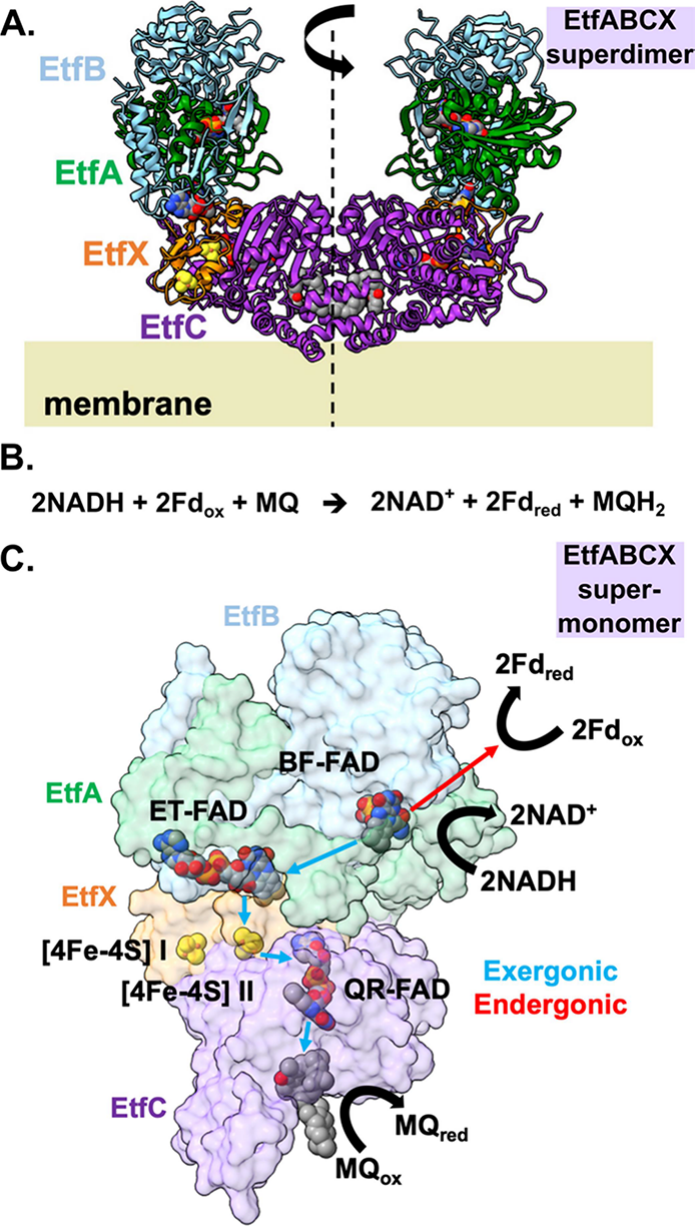
EtfABCX is a modular, membrane-associated superdimer of supermonomers, each possessing an independent, bifurcating EtfAB module. (A) Cryo-EM structure of a *T. maritima* EtfABCX superdimer with subunits labeled and color-coded, the 2-fold symmetric axis is shown, and membrane association depicted (PDB ID: 7KOE). (B) Overall reaction catalyzed by EtfABCX is shown. (C) Surface-represented EtfABCX supermonomer—rotated 90° relative to that shown in (A)—with cofactors, and the direction of electron flow during and after bifurcation is shown. Upon NADH oxidation, the endergonic branch (red arrow) of the bifurcation event sees an electron transferred to Fd, whereas electrons following the exergonic branch (blue arrows) transfer successively through ET-FAD, [4Fe–4S] II, and QR-FAD before their final donation to MQ. MQ is shown bound as in the cryo-EM structure.

We focus on the EtfABCX of the hyperthermophilic bacterium, *T. maritima*, which provides a model system suited for exploring BF mechanistically that benefits from high thermostability and recombinant expression in *Escherichia coli*.^13^ Originally identified from the mesophilic, N_2_-fixing bacterium, *Azotobacter vinelandii*, EtfABCX has also been studied in the hyperthermophilic, non-N_2_-fixing archaeon, *Pyrobaculum aerophilum*, which was heterologously expressed in *Pyrococcus furiosus*.^14^ The adoption by EtfABCX of a membrane-associated, superdimeric state sets it apart from other EtfAB-containing enzymes. Its 2.9 Å cryo-EM structure displays how its bifurcating subcomplex (EtfAB) is paired with its MQ oxidoreductase subcomplex (EtfCX).^10^ The low- and high-potential electrons are considered to follow endergonic and exergonic branches, respectively. In total, the EtfABCX superdimer possesses six FADs, four [4Fe–4S] clusters, and two MQs. EtfABCX oxidizes the two-electron donor NADH (reduction potential, or *E*_m_ = −320 mV) at the bifurcating flavin (BF-FAD) of EtfA before a low-potential electron donation to Fd (*E*_m_ = ∼−450 mV) and, at the terminal end of the exergonic pathway, a high-potential electron donation to MQ (*E*_m_ = −74 mV).^7,10,15^ Following the high-potential path post-BF, an electron is then transferred to the electron transfer flavin (ET-FAD) in EtfB, to one of two [4Fe–4S] clusters ([4Fe–4S] cluster II, the other being cluster I) in EtfX, to a quinone reductase FAD (QR-FAD) in EtfC, then to MQ in the binding pocket formed at the EtfC–EtfC interface.^10^

The overall reaction catalyzed by each EtfABCX supermonomer involves four electrons donated by NADH, two transiently interacting Fd molecules that become reduced by one electron each, and an MQ that accepts two electrons at the end of the cycle (Figure 1B). The catalytic cycle proposed based on the cryo-EM structure of EtfABCX and spectroscopic analyses posits two rounds of NADH oxidation and Fd reduction prior to MQ reduction and cycle completion. In the *P. aerophilum* ABCX, the ET-FAD of the high-potential pathway has an *E*_m_ of −94 mV for its anionic semiquinone (ASQ)/hydroquinone (HQ) couple, whereas the BF-FAD of the low-potential pathway has an *E*_m_ of −285 mV for its oxidized (OX)/HQ couple.^14^ These values indicate favorable ET steps with minimal energy dissipation, as they have reduction potentials higher than that of NADH (*E*_m_ = −320 mV) and are likely similar in EtfABCX. Alternatively, the OX/ASQ couple of BF-FAD is ∼−476 mV, a potential low enough to reduce Fd (*E*_m_ = ∼450 mV) as the terminal electron acceptor in the low-potential pathway. Moving from ET-FAD along the high-potential pathway, the [4Fe–4S] clusters in EtfX, with their identical coordination to the mammalian ETF ubiquinone oxidoreductase, likely have a reduction potential of ∼+37 mV and act as high-potential acceptors prior to reduction of MQ (*E*_m_ = −74) by QR-FAD.^10,16^

The conformational changes that underlie productive BF and ET in the system, if any, are thus far unobserved and could provide relevant insights into the mechanism.^9,10,12^ Each supermonomer of EtfABCX possesses a complete set of cofactors for catalysis and, in turn, encompasses the enzymatic machinery for transferring electrons from NADH to each of its low- and high-potential acceptors (Figure 1C). In EtfABCX, ET-FAD is bound to Domain II of EtfB, which is tethered to the core of EtfAB by two loops: one connecting Domains I and II of EtfB and the other being a C-terminal peptide of EtfA. A subclass of EtfABCX’s cryo-EM 3D classes was lacking density for portions of EtfB’s C-terminus, further hinting at flexible domain movements that enable interflavin ET between BF- and ET-FADs.^10^

In all available structures for EtfAB-containing BF-systems, the distance between BF-FADs and ET-FADs is too far apart (18–38 Å; Figure 1C) for productive ET.^9−12^ The distances observed and their incompatibility with electron transfer suggest that these systems possess a conformational malleability in the performance of their BF and ET catalysis—transitioning from transfer-capable intercofactor distances of less than 14 Å to the maximum observed of 38 Å. A mechanistic and cohesive description of the conformational changes required to transition between catalytic states from the available MX and cryo-EM structures is yet to be established. Throughout the known structures, the positioning and orientation of ET-FAD-binding domains dictate their respective FAD-FAD distances. In EtfABCX’s cryo-EM structure, the interflavin distance is 25 Å, designated as the C-state. In a hybrid experimental and computationally predicted structure, an EM result^17^ was combined with an AlphaFold2 model^18^ to produce a structure of the *A. woodii* Ldh-EtfAB complex where the flavins are 11.8 Å apart, representing a postulated bifurcation or B-state. A wholly experimentally derived example of a BF enzyme in the B-state is lacking. As a result, we hypothesize that BF- and ET-associated conformational changes in EtfABCX are coordinated with the cofactor redox state and coenzyme binding, conditions likely to reveal novel observable structural states that inform our understanding of the enzyme’s catalytic cycle.^10^

High-resolution detail in structural biology delivers a substantial component to our understanding of structure–function relationships of biological macromolecules but often fails to demonstrate how biologically relevant transition states, domain movements, and changes in conformation enable enzymatic reactions.^19,20^ As such, small-angle X-ray scattering (SAXS) is exquisitely suited to determine such behavior because macromolecular assembly is assessed in solution. Additionally, the available high-resolution structure for EtfABCX^10^ supplements the rotationally averaged information collected in SAXS. By combining SAXS with a high-resolution structure, we propose modifications to the structure that better fit the solution state.

One complication with using the *T. maritima* EtfABCX system as a model to investigate BF is its soluble propensity to exist as both a superdimer and supertetramer. Implementing size-exclusion chromatography in-line with multiangle light scattering, UV–visible spectroscopy, and SAXS (SEC-MALS-SAXS) circumvents this detail and allows for separate monitoring of protein molecular weight, cofactor redox state and occupancy, and solution structure, respectively.^21,22^ Furthermore, we have adapted the SEC-MALS-SAXS instrumentation to measurements under anaerobic conditions for the study of oxygen-sensitive metalloenzymes, including BF systems like EtfABCX.

Here, we reveal the conformational malleability of EtfABCX and elucidate the structural transitions between its BF- and ET-associated states using a novel assay combining synchrotron SEC-MALS-SAXS, multiwavelength UV–visible spectroscopy, an anaerobic sample environment, and rigid body modeling using SAXS data and molecular dynamics to sample protein conformational space.^21−24^ Our approach provides a platform for future anaerobic SEC-MALS-SAXS studies of BF systems, diverse oxidoreductase metalloenzymes, and other redox-sensitive systems reliant on domain motions. Applied here, this approach led to the identification of *T. maritima* EtfABCX solution states that are similar to static structures for homologous EtfAB-containing BF-enzymes and demonstrate bifurcation-like (B-like) and electron conducting-like (D-like) states. These states populate a conformational equilibrium that is similarly affected by NAD(H) binding and flavin reduction. We show that NAD(H) serves as a structural “trigger” for *T. maritima* EtfABCX’s conformational behavior, enabling transitions in domain positioning that couple redox cofactors for BF and ET. Each half of EtfABCX’s superdimer adopts independent conformational states, suggesting separate BF and ET processes whose high potential pathways converge at a shared site of MQ reduction. Our findings show the correlation between structural states and steps in the catalytic cycle of EtfABCX.

## Materials and Methods

### Production of Recombinant EtfABCX and EtfAB

Recombinant forms of *T. maritima* EtfABCX and EtfAB were obtained from *E. coli* as previously described.^13^ In brief, PCR amplicons of the *T. maritima* EtfABCX operon were cloned into the pET-21a(+) plasmid (Novagen, Merck KGaA, Darmstadt, Germany) with an N-terminal 9× His tag and transformed into an *E. coli* BL21 (DE3) Δ*iscR* strain with plasmid pLysS (Novagen, Merck KGaA, Darmstadt, Germany). The expression strain was cultured anaerobically at 37 °C in LB media supplemented with 100 μM ferrous ammonium sulfate, 100 μM cysteine, 0.5% (w/v) glucose, 0.5% (w/v) fumarate, and 0.1 mg/L riboflavin, kanamycin (50 μg/mL), and chloramphenicol (30 μg/mL) in a 20 L fermenter. Recombinant protein production was induced at an OD_600_ ∼ 0.6 by the addition of 0.5 mM IPTG and temperature was reduced from 37 to 30 °C. Cells were harvested after 16 h and both EtfABCX and EtfAB were purified from the cytoplasmic fraction by anaerobic chromatography using a HisTrap FF column (Cytiva, Marlborough, MA, USA) and Superdex 200 HiLoad 26/60 column (Cytiva, Marlborough, MA, USA). EtfAB protein concentrations were estimated by the Bradford assay, while the EtfABCX protein concentration was first estimated by the Bradford assay and then corrected by the results from an amino acid analysis (AAA Service Laboratory, Inc., Boring, Oregon, USA).^25^ Quantifications of FAD and Fe were described by Ge et al. For FAD quantification, proteins were denatured by 1% SDS at room temperature to release FAD, and an extinction coefficient of 11.3 mM^–1^ cm^–1^ was used to measure FAD. Fe was measured using a bathophenanthroline colorimetric assay. The samples used herein contained approximately three and two molecules of FAD per EtfABCX supermonomer and EtfAB heterodimer, respectively, and eight and zero Fe atoms per EtfABCX supermonomer and EtfAB heterodimer, respectively, in accordance with the cryo-EM structure of EtfABCX.^10^

### Detergent Screening with DLS

EtfABCX was prepared at 6 mg/mL in standard buffer containing 20 mM Tris-HCl, pH 8.0, 200 mM NaCl either with or without varying concentrations of CHAPS, LDAO, Triton X-100, Tween-20, and DDM detergents before transfer into a 96-well plate and measured for DLS with a DynaPro Plate Reader III (Wyatt Technology, Goleta, CA, USA). Data were analyzed by using DYNAMICS software (Wyatt Technology, Goleta, CA, USA) before export. Representative measurements were plotted by % number versus *R*_h_ for presentation in the supplement.

### SEC-MALS-SAXS Data Collection and Processing

Purified EtfABCX and EtfAB proteins were thawed in an anaerobic glovebox (Coy Laboratory Products, Grass Lake, MI, USA) maintained in an inert gas atmosphere (95% N_2_, 5% H_2_) after which they were diluted using standard buffer either with or without 0.01% DDM, respectively, and in the presence or absence of a reductant or coenzyme. All samples in this study have their compositions detailed in Table S1. Buffers were made anaerobic through three cycles each of alternating N_2_ purging (15 min) and vacuum pumping (15 min) using a Schlenk line apparatus, after which they were stored either within a glovebox or under positive pressure with N_2_ to finish equilibrating toward anaerobicity. Samples were sealed anaerobically in a 96-well plate prior to injection into the anaerobically equilibrated SEC-MALS-SAXS system at the Structurally Integrated BiologY for Life Sciences beamline (SIBYLS, BL 12.3.1) of the Advanced Light Source (ALS) located at Lawrence Berkeley National Laboratory (LBNL).^21,22,26^ A 1290 Infinity II series high-performance liquid chromatography (HPLC) system (Agilent, Santa Clara, CA, USA) with an autosampler was used for sample injection and SEC. Autosampler and column temperatures were equilibrated to room temperature before analyses began. The anaerobic running buffer was maintained under positive pressure by using inert gas. The beamline was configured to a 1.24 Å X-ray wavelength and 2075 mm sample-to-detector distance to obtain the relevant wave vector transfer, *q* = 4πsin(θ)/λ, where 2θ is the scattering angle and λ is the X-ray wavelength, yielding a *q*-range from 0.01 to 0.4 1/Å.^21^ A KW-803 column (Showa Denko, Tokyo, Japan), selected for its optimal separation of EtfABCX species, was equilibrated with an anaerobic standard buffer containing 1 mM dithiothreitol for at least six h to scrub the silica-based column of residual O_2_ before re-equilibration to normal standard buffer. SEC was performed using a flow rate of 0.65 mL/min during 2 s X-ray exposures over the course of 25 min SAXS data collections, wherein a PILATUS3 × 2 M Detector (Dectris, Baden, Switzerland) was used to record images. SAXS images were radially integrated before being background subtracted using BioXTAS RAW (RAW), after which subtracted SAXS profiles were merged and used for analysis in the same software.^27^ Some SEC-SAXS data sets required the application of a linear baseline correction in RAW to account for shifting baselines in the SAXS signal due to capillary fouling. The performance of SEC-SAXS’s separation of EtfABCX peaks combined with software-assisted singular value decomposition evolving factor analysis (SVD-EFA) ameliorated residual scattering contributions from the supertetramer and allowed obtainment of the NADH-reduced superdimer’s SAXS profile.^28^

MALS experiments were performed under anaerobic conditions using an 18-angle DAWN HELEOS II light scattering detector connected in tandem to an Optilab T-rEX differential refractive index detector (Wyatt Technology, Goleta, CA, USA), both in-line with the SEC-SAXS system. System normalization and calibration were performed using bovine serum albumin (BSA) with 55 μL injections at 7 mg/mL in buffer matching that of each sample. MALS data analysis was performed using Astra 8 software (Wyatt Technology, Goleta, CA, USA), where d*n*/d*c* values were obtained and MALS MW values were determined from the primary SEC peaks of each sample.

In-line UV–visible spectra were measured during each SEC-MALS-SAXS run in order to monitor the cofactor redox state and occupancy through the use of an in-line Diode Array Detector (Agilent, Santa Clara, CA, USA) as part of the 1290 Infinity II series instrument. Absorbance was measured at 280, 374, 390, 454, and 636 nm to monitor the protein, cofactor, and charge-transfer complex behaviors. UV–visible spectral data were exported from Agilent ChemStation software before their normalization to the *A*_280_ peak maxima of either the EtfABCX superdimer or EtfAB heterodimer reference specimen to correct for increased background from coenzyme presence or to correct for changes in superdimer–supertetramer populations in some conditions. Static, batch-mode UV–visible spectra were measured on exemplar specimens prior to SEC-MALS-SAXS with an N60 Mobile UV/vis spectrophotometer (Implen, Westlake Village, CA, USA).

### SAXS Data Analysis and Modeling

Background-subtracted SAXS profiles were analyzed in RAW for Guinier analyses, generating *P*(*r*) plots, and for performing Kratky analyses.^29−31^*P*(*r*) plots were normalized by their forward scatter (*I*(0)). SAXS MW calculations, also calculated in RAW, were based on the volume of the correlation (*V*_c_) method,^32^ which inherently carries the potential for 5–10% error. Theoretical scattering profiles were generated using the Debye formula with FoXS.^33−35^ SAXS profiles used in figures were sometimes scaled for the sake of clarity of presentation. Volume calculations were performed in ScÅtter (https://bl1231.als.lbl.gov/scatter/). Plotting of data and statistical analyses were performed in Prism (GraphPad, Boston, MA, USA).

Conformational sampling, scoring of conformers with the SAXS data, and enumeration of any resultant multistate models of EtfAB were performed using BilboMD.^23^ The EtfAB subcomplex containing BF-and ET-FADs was prepared for input to BilboMD using CHARMM-GUI’s PDB Reader and Manipulator.^36,37^ The ET-FAD-binding Domain II of EtfB allowed diffuse mobility with respect to the EtfAB core, as tethered by flexible loops connecting rigid bodies. This configuration was chosen based on a comparison of *T. maritima* EtfAB’s structure to that of *C. difficile* EtfAB-Bcd and *A. woodii* CarCDE. 800 conformers were generated within ±7.5 Å of the experimental reciprocal space *R*_g_s. The conformers were scored against the SAXS data before multistate ensembles were enumerated. The top-scoring ensembles were selected based on χ^2^ and their improvement over input as well as other multistate models.

The high-resolution structure of EtfABCX (PDB ID: 7KOE) was also used for the flexible refinement modeling approach of SREFLEX, part of the ATSAS package.^38,39^ SREFLEX uses NMA to estimate flexibility within proteins and improves their fit to experimental data through flexible refinement. EtfABCX’s structure was segmented based on subunit composition before input to SREFLEX. Models from the restrained refinement stage with the highest agreement to the scattering data (lowest χ^2^) as well as those with the lowest clash and breaks values were selected for structural comparison to the cryo-EM model as shown in the supplement.

SAXS data sets for this study were deposited into the Simple Scattering open data repository (https://simplescattering.com/) with the data set code XSDUNFBU.^40^

## Results

### Isolation of EtfABCX Superdimer for Biophysical Analysis with Anaerobic SEC-MALS-SAXS

To test whether conformational mailability could be detected in solution by SAXS, we first attempted to replicate the conditions and assembly measured in cryo-EM and compare the experimental SAXS signal against the calculated SAXS signal that would be attained from a cryo-EM-like structure. A significant fraction of EtfABCX molecules adopts a supertetrameric state (EtfABCX_2_–EtfABCX_2_) along with the superdimer. The supertetramers were observed but not analyzed in detail in cryo-EM and were reportedly decreased by adding detergent to solutions prior to analysis. While the multimers can be distinguished by SEC, they are in transient equilibrium and the trailing edge of the supertetramer elution overlaps with the leading edge of the superdimer. Maximizing the superdimer population relative to the supertetramer improves the derived signal of SEC-SAXS and reduces complications in the analysis of mixed species. A detergent screen was performed by using dynamic light scattering (DLS) to assess polydispersity and changes in hydrodynamic radii (*R*_h_) to identify optimal solution conditions for analyzing the superdimer (Figure S1). Polysorbate 20 (Tween-20) and *n*-dodecyl-β-D-maltoside (DDM) surfactants showed appreciable minimization of supertetramer populations at concentrations below their critical micelle concentrations (CMCs), but the latter was adopted due to the former detergent’s proclivity to oxidatively damage proteins due to residual peroxide impurities.^41,42^

**Figure 2:**
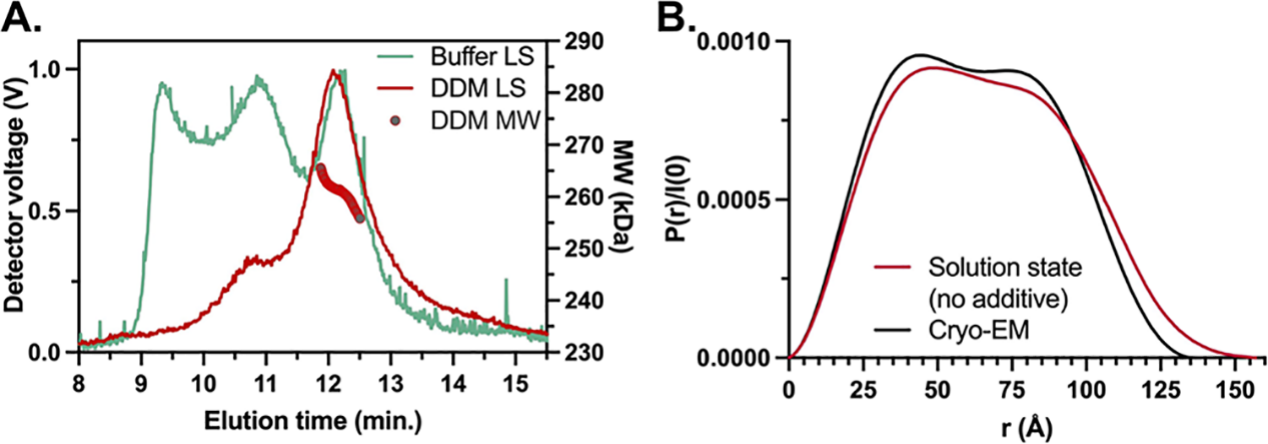
SEC-MALS-SAXS analysis of EtfABCX.(A) SEC-MALS light scattering (LS) traces showing the minimization of supertetramer and aggregate species of EtfABCX in 0.01% DDM-containing running buffer (DDM LS) as opposed to the buffer only condition (Buffer LS). MW values of species in the superdimer peak obtained from MALS are as expected for the 257 kDa, 9× His-tagged EtfABCX (DDM MW). (B) *P*(*r*) plots calculated from EtfABCX’s SAXS profile and that computed from its cryo-EM structure show that EtfABCX’s supermonomer halves become less correlated in solution. *P*(*r*) plots were normalized by *I*(0) for ease of comparison.

Time-dependent, oxygen-induced aggregation was observed during initial exploration with air-exposed samples of EtfABCX and thus serves as a litmus test for oxygen encroachment during SEC-MALS-SAXS experiments (Figure S2). To preserve cofactor redox states and avoid aggregation, all SEC-MALS-SAXS experiments were conducted under anaerobic conditions to preserve physiologically relevant and mechanistically productive behavior. Anaerobicity was maintained throughout sample preparation in an anaerobic chamber containing an inert gas atmosphere (95% N_2_, 5% H_2_) and by injection into SEC-MALS-SAXS instrumentation equilibrated with an anaerobic running buffer. A standard buffer containing 0.01% DDM was used during all anaerobic SEC-MALS-SAXS experiments of EtfABCX and showed appreciable minimization of aggregate and supertetramer species as well as isolation of the superdimer (Table S1, Figures 2A, and S3, S4A). The MALS-derived molecular weight (*M*_W_ ∼ 260 kDa) for the superdimer peak was identical to the MW for the 9× His-tagged complex (257 kDa; Table 1, Figures 2A, and S4A,B) and validates the superdimeric state in solution.

**Table 1:** SEC-MALS-SAXS Parameters for EtfABCX

sample	condition	Guinier *R*_g_, Å	IFT *R*_g_, Å	*D*_max_, Å	MALS *M*_W_, kDa
EtfABCX	no additive	50.2 ± 0.7	50.7 ± 0.1	157	265
	NADH	48.9 ± 0.4	50.7 ± 0.2	154	266
	NAD^+^	47.8 ± 1.2	48.8 ± 0.4	144	255
	DT	51.2 ± 0.4	50.3 ± 0.1	156	248

### Comparison of EtfABCX Solution Conformation with the Available High-Resolution Cryo-EM Structure

Analysis of EtfABCX’s main elution peak from SEC-MALS-SAXS yields results that are consistent with the superdimer assembly but indicate that the complex is in a different conformation than that reported in the cryo-EM structure. The radius of gyration (*R*_g_) was measured to be 50.2 Å and the mass was measured to be consistent with the 257 kDa complex (Table 1 and Figure S5A).^29,43^ However, the calculated SAXS curve for the cryo-EM structure of the EtfABCX superdimer (PDB ID: 7KOE) varies from the solution state experimental SAXS data with a χ^2^ of 3.35 (Figure S6).

A more intuitive comparison comes from the resulting *P*(*r*) function (a Fourier transform of the scattering data) that describes the interelectron distances from within a molecule.^44^ The *P*(*r*) function from the 2-fold symmetric cryo-EM “U”-shaped structure (Figure 1A) has two nearly equivalent peaks (Figure 2B). Starting from short length scales, the first peak in the *P*(*r*) function at 45 Å is dominated by all of the interelectron distances populating the cross-section of the length of the “*U*”. The second distinct peak at 80 Å represents the correlated distances between the supermonomer arms. The maximum dimension (*D*_max_) of the cryo-EM structure’s *P*(*r*) at 135 Å is defined by a dramatic decay to zero. The two peaks from the experimental *P*(*r*) are less distinguishable and weighted toward the shorter distance cross-section peak. The experimental *D*_max_ is approached more gradually with a larger *D*_max_ of 157 Å. These features suggest a loss of symmetry, an elongation of the structure, and changes in the spatial correlation of the supermonomers, which we model in detail below. Having observed differences between the available cryo-EM structure of the superdimer and the measured SEC-SAXS result, we took a divide-and-conquer approach starting with the EtfAB subcomplex and later connected these results to EtfABCX.

### Solution Conformation of EtfAB and the Impact of Coenzyme Binding and Cofactor Reduction

**Figure 3:**
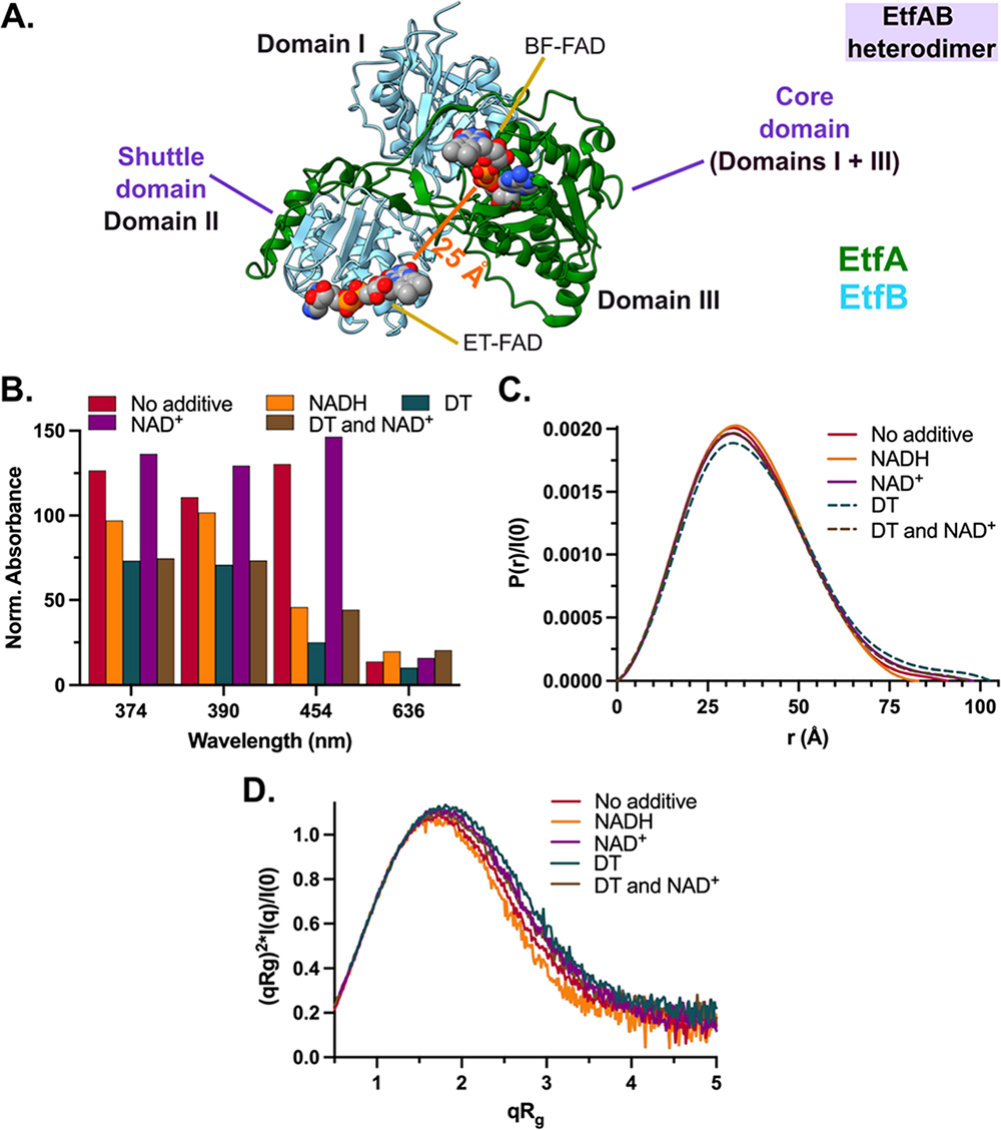
Effects of NAD(H) binding and flavin reduction on EtfAB as monitored by optical spectroscopy and SAXS. (A) Bifurcating module of EtfABCX, EtfAB (PDB ID: 7KOE; EtfA in green, EtfB in light blue), contains a shuttle domain (Domain II, EtfA and EtfB; ET-FAD-binding) flexibly tethered to its core domain (Domains I and III, belonging to EtfB and EtfA, respectively; BF-FAD-binding) via loops connecting Domains I and II of EtfB and a C-terminal peptide of EtfA. The FAD-FAD distance for the C-state from the cryo-EM structure is shown (bold orange). Domains are labeled in bold, whereas cofactors are labeled in a normal font. (B) Absorption profiles for EtfAB in various running buffer conditions were measured during SEC-MALS-SAXS to monitor the flavin redox state, each measurement normalized to the *A*_280_ peak maximum of the “No additive” condition’s EtfAB heterodimer SEC peak. The peak maximum for each recorded wavelength is compared here. (C) *P*(*r*) functions were obtained from merged SEC-SAXS profiles and normalized by *I*(0) for each condition shown in (B). (D) EtfAB’s SAXS data, shown as dimensionless Kratky plots, show varying degrees of flexibility resulting from flavin reduction and coenzyme binding. The NADH-reduced condition is considered the least flexible and compact, whereas the DT-reduced condition represents the opposite. NAD^+^ and NAD^+^/DT conditions are highly overlapping.

The EtfAB subcomplex of EtfABCX provides a simplified heterodimer system in which to study BF and ET without heterogeneity in assembly (Figure 3A). EtfAB also retains the ability to undergo the chemical reduction of its flavins. One feature of this system that was previously discovered is that, upon purification under anaerobic conditions, it retains a one-electron, ASQ state with respect to its ET-FAD, so we will refer to the enzyme assuming it is “as-purified” unless otherwise stated.^13,14^ As such, we measured anaerobic SEC-SAXS and UV–visible spectroscopy of EtfAB in running buffers containing no additive or containing excess quantities of NADH, NAD^+^, DT, or a DT/NAD^+^ mixture (Tables 2, S1, Figures 3B and S5E–I). SAXS MW values, as determined using the volume of correlation method,^32^ indicate the data to reflect monodisperse solutions of EtfAB heterodimer (Table 2).

**Table 2:** SEC-SAXS and Multi-State Model Parameters for EtfAB

		primary analysis				multistate models							
						compact state			extended state			χ^2^	
sample	condition	Guinier *R*_g_, Å	IFT *R*_g_, Å	*D*_max_, Å	SAXS *M*_W_, kDa (69 Exp.)	*f*_compact_	*R*_g_, Å	*D*_max_, Å	*f*_extended_	*R*_g_, Å	*D*_max_, Å	one-state	two-state
EtfAB	no additive	27.1 ± 0.1	27.5 ± 0.1	91	63	0.39	25.1	74	0.61	28.1	88	1.41	1.09
	NADH	26.6 ± 0.2	27.0 ± 0.1	79	64	0.85	25.8	82	0.15	26.5	85	0.87	0.85
	NAD^+^	27.9 ± 0.2	28.2 ± 0.1	98	64	0.59	26.3	83	0.41	30.3	105	1.52	1.08
	DT	28.9 ± 0.1	29.5 ± 0.1	103	65	0.65	25.9	82	0.35	32.9	110	1.63	1.16
	DT and NAD^+^	27.5 ± 0.1	28.1 ± 0.1	97	60	0.75	26.2	85	0.25	31.5	103	1.28	1.00

In-line UV–visible spectra recorded during SEC elution monitored absorbances at 280, 374, 390, 454, and 636 nm to assess changes in the cofactor redox state. Specifically, 280 nm allows tracing of protein content; 374, 390, and 454 follow FAD occupancy and redox state; and changes at 636 nm are indicative of charge-transfer complex formation. Absorbance measurements of EtfAB in the presence of excess NADH indicated that its FAD cofactors were fully reduced (Figures 3B and S8E,F). The *P*(*r*) functions (Figure 3C) and *R*_g_ values (Table 2) indicate a more compacted state upon reduction with NADH and, in turn, suggest an increase in the proximity of BF- and ET-FADs. Dimensionless Kratky plots^30^ (Figure 3D) show decreased flexibility of EtfAB upon reduction with NADH relative to its resting state. Alternatively, EtfAB in the presence of NAD^+^ led the enzyme into a more extended state with increased flexibility (Table 2, Figures 3B–D, and S8G) with the *R*_g_ and *D*_max_ values exceeding the no additive and NADH-reduced conditions. To separate the effects of FAD reduction from NAD(H) binding, EtfAB was reduced with DT and shown to exhibit an extended state with increased flexibility (Table 2, Figures 3B–D, and S8H). The addition of NAD^+^ to the DT-reduced enzyme saw a reversion toward the NAD^+^-only condition with respect to structural features even though the enzyme remained reduced (Table 2, Figures 3B–D, and S8I). Taken together, NADH induces a compact rigid conformation, while NAD^+^ promotes greater flexibility and more extended conformations.

**Figure 4:**
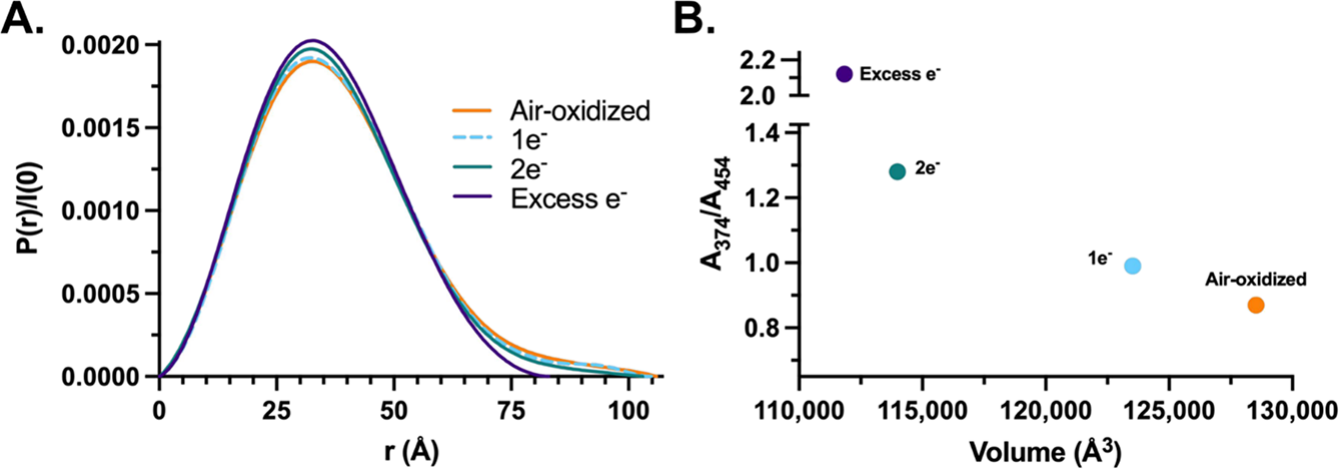
Structural features of EtfAB with its FADs in different redox states upon reduction with varying equivalents of NADH. (A) Distance distribution functions obtained from SEC-SAXS data. *P*(*r*) plots are normalized by *I*(0) for ease of comparison. (B) Ratios of absorption at *A*_374_ and *A*_454_ for EtfAB’s FADs and the corresponding molecular volumes for each condition are shown in (A).

In order to examine EtfAB with BF- and ET-FAD in different redox states, we measured the enzyme prepared as “air-oxidized” or in the presence of 0, 1, 2, or excess electron (e^–^) equivalents NADH by first exposing the enzyme to air to completely oxidize them before their return to anaerobicity and subsequent preincubation with 0, 0.5, 1, or 10 equiv NADH, respectively (Table S2 and Figure S10A–D). Comparing *P*(*r*) functions (Figure 4A), it is clear that reduction of BF- and ET-FAD leads to compaction between EtfAB’s core and shuttle domains as reflected in the increasing interelectron distance peak’s height along with decreasing *D*_max_ values (Table S2). A similar relationship is seen between *A*_374_/*A*_454_ absorption ratios and molecular volume, these wavelength bands being sensitive to FAD’s redox state and higher ratios indicating reduction (Figure 4B).

### Characterizing the Conformational Equilibria of EtfAB

To connect the SAXS experiments on EtfAB to high-resolution structural detail, we used a molecular-dynamics-based approach because of the obvious SAXS indicators of increased flexibility. Using the program BilboMD,^23^ we allowed flexibility in the ET-FAD-binding shuttle domain (Domain II; Figure 3A) based on variations observed in available high-resolution structures of homologues.^10−12,17^ BilboMD provides the best fitting conformations to the SAXS data. In addition, a weighted combination of two-, three-, and four-state conformations is also tested for comparative improvements to identify the minimal ensemble required to fit the data.

**Figure 5:**
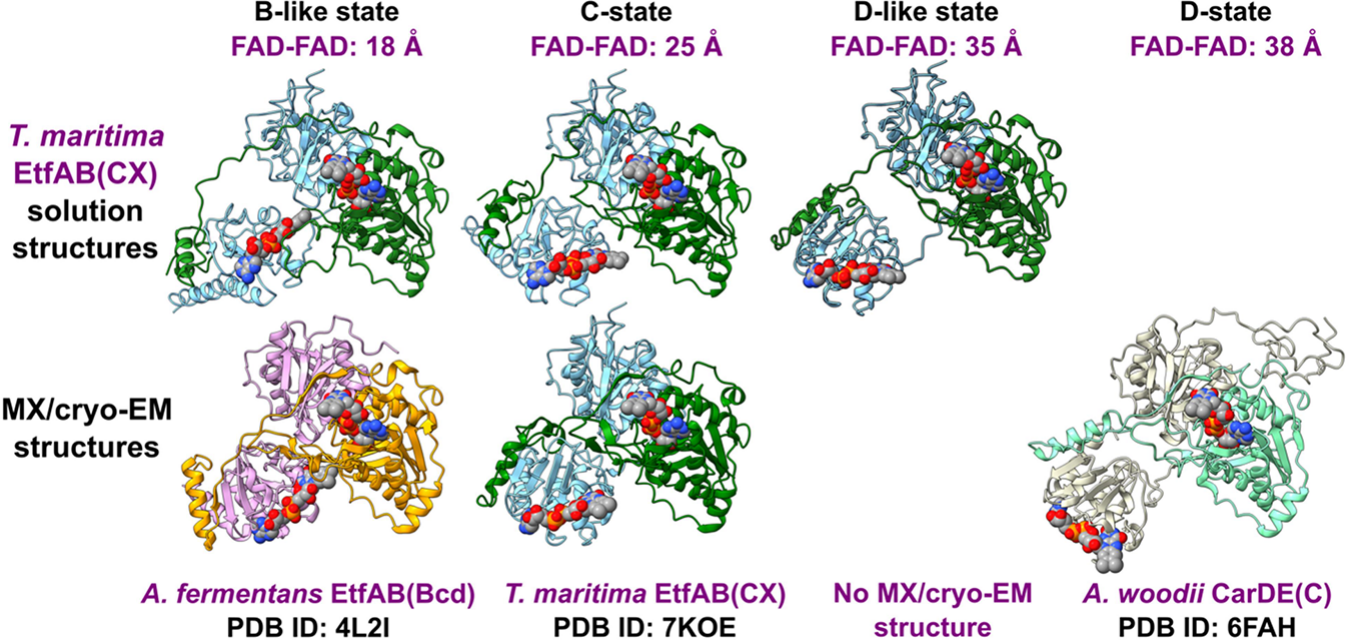
EtfAB’s conformational equilibrium includes conformations recapitulating the catalytic states observed in homologous BF-enzymes and a novel conformation. Representative solution structures of *T. maritima* EtfAB (top row; this study) identified in one- (C-state) and two-state equilibria (B-like and D-like states) are compared to their static counterparts from homologous BF enzymes (bottom row), revealing conformational states present during catalysis. FAD–FAD represents the end-to-end distances of BF- and ET-FADs in the models shown. No static structure for the D-like state; likewise, a D-state molecule with identical FAD-FAD distances and domain orientations was not identified. No B-state conformation has been identified from any experimental results.

All experimental conditions were fit with two-state models, and additional conformations provided no improvement. The optimal outputs included conformations that closely resembled high-resolution models determined from homologous EtfAB-containing BF-enzymes but are yet to be observed in *T. maritima* EtfAB (Table 2, Figures 5, and S11A–E). The conformations fell into three general categories: the C state with an overall conformation resembling the cryo-EM structure of *T. maritima* EtfAB and with end-to-end inter-FAD (FAD–FAD) distances of 25 Å; the B-like state (bifurcation-like, as in the *A. fermentans* EtfAB), with ET-FADs pivoting such that their isoalloxazine rings project toward BF-FAD with FAD-FAD distances of 18 Å; and the D-like state, here referred to as “electron conducting state” and resembling the previously reported dehydrogenase conducting state (D-states of *A. woodii* CarDE and *C. difficile* EtfAB with FAD–FAD distances of 38 and 37 Å, respectively), in which its conformation is quite extended and the inter-FAD distance is 35 Å. Examples of these conformations are shown in Figure 5 and taken from BilboMD outputs to fit EtfAB-NAD^+^ (Figure S11C). When restrained to a single conformation, the best model for all conditions was a C-state-like conformation. The C-state places EtfA and EtfB at an intermediate distance from one another, between the B- and D-like states. When two-state models improved the quality of the fit, the two conformations chosen as contributing to the best fit were B-like and D-like states in varying weighted proportions (Table 2 and Figure S11).

Using the percentages of B-like and D-like states, we estimate the conformational equilibrium among conditions. In the no additive condition, EtfAB exists in conformational equilibrium with 39% of its population in compact states and 61% in extended states, or with a *K*_eq_ (*K*_eq_ = [compact]/[extended]) of 0.64 (Table 2). Upon reduction by NADH, EtfAB’s *K*_eq_ shifts to 5.66 with 85% of its population in a compact state. The modest increase in χ^2^ between one- and two-state models for this condition is likely due to NADH-reduced EtfAB primarily existing in a compact state, the most parsimonious description having interflavin distances somewhere between those in B-like and C-states (18–25 Å). Alternatively, the presence of NAD^+^ shifts the equilibrium toward a more extended degree (see *R*_g_ values in Table 2) but intermediate in fraction extended relative to that seen in other conditions (*K*_eq_ = 1.44). This condition, along with the nonadditive one, both saw improvement upon fitting a two-state model to explain their conformational equilibria. DT-reduction of EtfAB saw a shift to a *K*_eq_ value of 1.86, with more of the population exhibiting compaction than the no additive or NAD^+^ conditions but some of the population extending more significantly (Table 2; see real space *D*_max_ and *R*_g_ values). Mixing DT and NAD^+^ with EtfAB led to a more compact state, not quite as compact as that seen after NADH treatment but more so than the other conditions. These trends are reflected in the *P*(*r*) and Kratky plots (Figure 3C,D).

### Characterizing the Conformational Changes of EtfABCX in Varying Redox States and in the Presence of Cofactors

**Figure 6:**
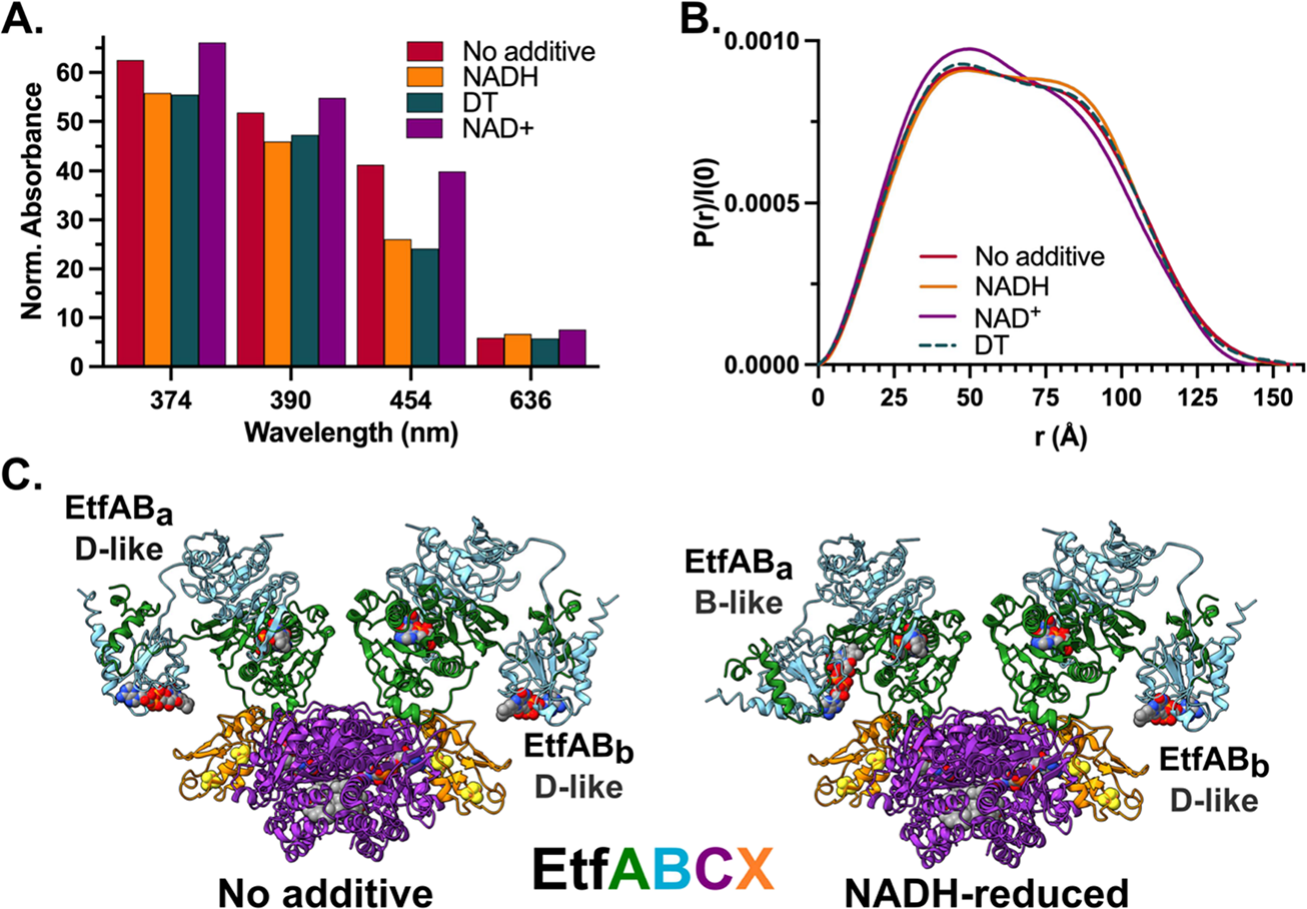
EtfABCX’s conformational states are impacted by cofactor reduction and coenzyme binding.(A) Absorbance traces for EtfABCX in various running buffer conditions were measured during SEC-MALS-SAXS to monitor the flavin redox state, each measurement normalized to the *A*_280_ peak maximum of the “No additive” condition’s EtfABCX superdimer SEC peak. The peak maximum for each recorded wavelength is compared here. (B) *P*(*r*) functions were obtained from merged SEC-SAXS profiles and normalized by *I*(0) for each condition shown in (A). (C) Best-fitting model of EtfABCX superdimer in the “No additive” condition with EtfAB modules (EtfAB_a_, left of the complex; EtfAB_b_, right of the complex) in conformations obtained from modeling of heterodimeric EtfAB is shown (left). Superdimers are rotated 45° for clarity. Similarly, a best-fitting model of EtfABCX superdimer in the “NADH-reduced” condition with EtfAB modules in conformations similarly obtained from modeling of heterodimeric EtfAB fits is shown (right).

Having observed conformational changes of EtfAB in the presence of cofactors and varied redox states, we tested whether similar mixtures induced changes in EtfABCX. Upon reduction of BF-FAD by the physiological electron donor, NADH, we measured SEC-MALS-SAXS along with UV–visible spectra of EtfABCX in a running buffer containing 10 equiv (Eq) of NADH per EtfAB subcomplex in the superdimer (Table 1, Figures 6A, and S4A,B, S5). In-line UV–visible spectra recorded during SEC elution monitored absorbances at 280, 374, 390, 454, and 636 nm to assess changes in the flavin and iron–sulfur cluster redox state (Figures 6A and S8A,B, S9). The observed decrease in absorbance in the presence of NADH at 374, 390, and 454 nm is indicative of BF- and ET-FAD reduction while the modest increase at 636 nm suggests the presence of an EtfABCX-NADH complex. Given that NADH was present at a 10-fold excess over EtfABCX, the BF- and ET-FADs are assumed to be in hydroquinone states, that is, fully reduced. The scattering data on NADH-saturated EtfABCX interestingly resulted in a *P*(*r*) function with more correlated distances between EtfABCX, arms suggesting a nearly fully symmetric state, as shown in the cryo-EM structure (Figures 6B and S5B).

In addition to the observed changes in the conformation of the superdimer in the NADH-reduced state, an increase in the supertetramer population was observed during anaerobic SEC elution, keeping detergent concentrations consistent (Figures S4A and S4B). In grids used in the cryo-EM studies, a supertetramer was observed but not characterized at high resolution.^10^ Put roughly, the supertetramer has an “X” shape where two “U”-shaped superdimers are stacked bottom to bottom, making contacts at surfaces of EtfC (Figure S3C). The calculated scattering from a model created in this way matches the SAXS data (Figure S3D). EtfC has been postulated to be the major contact for membrane association, as it holds hydrophobic MQs. Taking the above behavior together, the symmetric form of EtfABCX exposes a more hydrophobic patch on EtfC and forms the basis for symmetric multimerization.

The role of NAD^+^ binding to EtfABCX was investigated to determine its effects free from those of flavin reduction (Table 1, Figures 6A,B, and S5C, S8C). Relative to the no additive condition, addition of NAD^+^ showed a modest increase in absorbance at 374, 390, 454, and 636 nm, likely due to the formation of an EtfABCX-NAD^+^ complex (Table 1 and Figure 6A). Analysis of the scattering data yielded a *P*(*r*) function with a greatly diminished correlation between supermonomers, indicative of a more extreme asymmetry for this state (Figures 6B and S5C). Full DT-reduction of the system in the absence of NADH or NAD^+^ yielded the same results as the apo system (Table 1 and Figure 6A,B). This was the case even though the enzyme’s absorbance indicated a reduced state (Figures 6A and S8D). In further disagreement with the NADH-reduced state, reduction by DT did not lead to an increase in the supertetramer content (Figure S4C).

Summarizing the above findings on EtfABCX, NAD^+^ and NADH cause the greatest conformational differences. In the presence of NAD^+^, EtfABCX is in the most asymmetric state, while with NADH, EtfABCX is in the most symmetric state. In the NADH state, a larger percentage of EtfABCX forms supertetramers. The NADH-saturated superdimers are in the best agreement with the high-resolution cryo-EM structure.

### Atomistic Modeling of EtfABCX’s Conformational States

To provide a connection to the conformational changes that may be occurring for the observed differences between the solution SAXS data and the cryo-EM results (Figure 1A), atomistic models for EtfABCX were constructed. These models were comprised of EtfAB subunits in varying combinations of the states obtained through BilboMD’s conformational sampling of EtfAB alone (B-like, C-, or D-like states). The calculated SAXS profiles of the models were compared to experimental data to determine which states, if any, best characterized the enzyme under different conditions (Figures 5 and 6C). The highest-scoring models (Figure S12) were all in possession of at least one supermonomer whose EtfAB component was in an extended, D-like state, while the other supermonomer was in either the B-like (NADH-reduced) or C-like states (no additive). The excess NAD^+^ condition elicited a conformation for EtfABCX with one EtfAB module in a compacted form resembling the C-state and the other module remaining in a D-like state (not shown).

To test whether an alternative modeling approach would improve upon the highest-scoring models described above, we applied a normal-mode analysis-based (NMA) flexible refinement of the cryo-EM structure using SREFLEX.^38,45^ The best-fitting model was one of an EtfABCX superdimer in which its supermonomers were twisted about their EtfC–EtfC interface (Figure S7A). The angles calculated between BF-FAD, ET-FAD, and [4Fe–4S] II clusters from each supermonomer were 162° and 128° (162°/128°) compared to 138°/138° in those of the cryo-EM result. Changes were observed for the ET-FAD and [4Fe–4S] cluster II intercofactor distances, placed at 22 and 7.5 Å separation on each respective supermonomer. This contrasts with the 12.3 Å distances seen in the symmetric input structure (Figure S7B) and is partially the result of the movement of EtfX subunits’ movement. Using this method, the distances between BF-FAD and ET-FAD remained the same as in the cryo-EM structure. Similar modeling with SAXS data under the NADH and NAD^+^ conditions yielded similar conformations and varied only slightly in the aforementioned structural parameters (not shown). The more asymmetric conformation improves the quality of fit, with a χ^2^ below a value of 2.1 (Figure S7C), indicating that the model fits the data to within error in contrast to the cryo-EM result (Figure S6). Although both NMA- and BilboMD-generated models fit the data to a reasonable degree, comparing the no additive condition’s *P*(*r*) functions from primary analysis and that of the BilboMD-based model shows superior agreement with the NMA-refined model’s *P*(*r*) function (Figure S13).

The superiority of the BilboMD model is further supported by multiple lines of evidence. Not only does the model fit the data better but also the model stems from results on the subcomplex where SAXS signal differences are attributed to the motion of fewer pieces. Furthermore, this result provides biological insight in that the increased flexibility from a D-like state allows interactions between ET-FAD and the [4Fe–4S] II cluster in EtfX, as discussed below.

## Discussion

### Identification of Conformational States and Their Relation to Catalytic Steps

While the static structure of *T. maritima* EtfABCX is foundational for understanding the mechanism, the structure cannot explain productive BF and ET states. In this study, we set out to test, identify, and trigger the EtfABCX mechanics that make BF possible and are suggested by homologues in alternate conformations. A proposed catalytic cycle for EtfAB-containing BF-enzymes was recently published alongside the high-resolution model for EtfABCX.^10^ The *T. maritima* cryo-EM structure revealed a novel C-state in which the two FAD cofactors in EtfAB are 25 Å apart and placed this state intermediate to the previously observed B-like (18 Å apart; “compact”) and D-states (38 Å apart; “extended”) of *A. fermentans* EtfAB and *A. woodii* CarDE, respectively, alongside a hypothetical BF (B) state.^9,11^ In this study, we provide experimental observations supporting the existence of the hypothesized conformational changes by EtfABCX.

**Figure 7:**
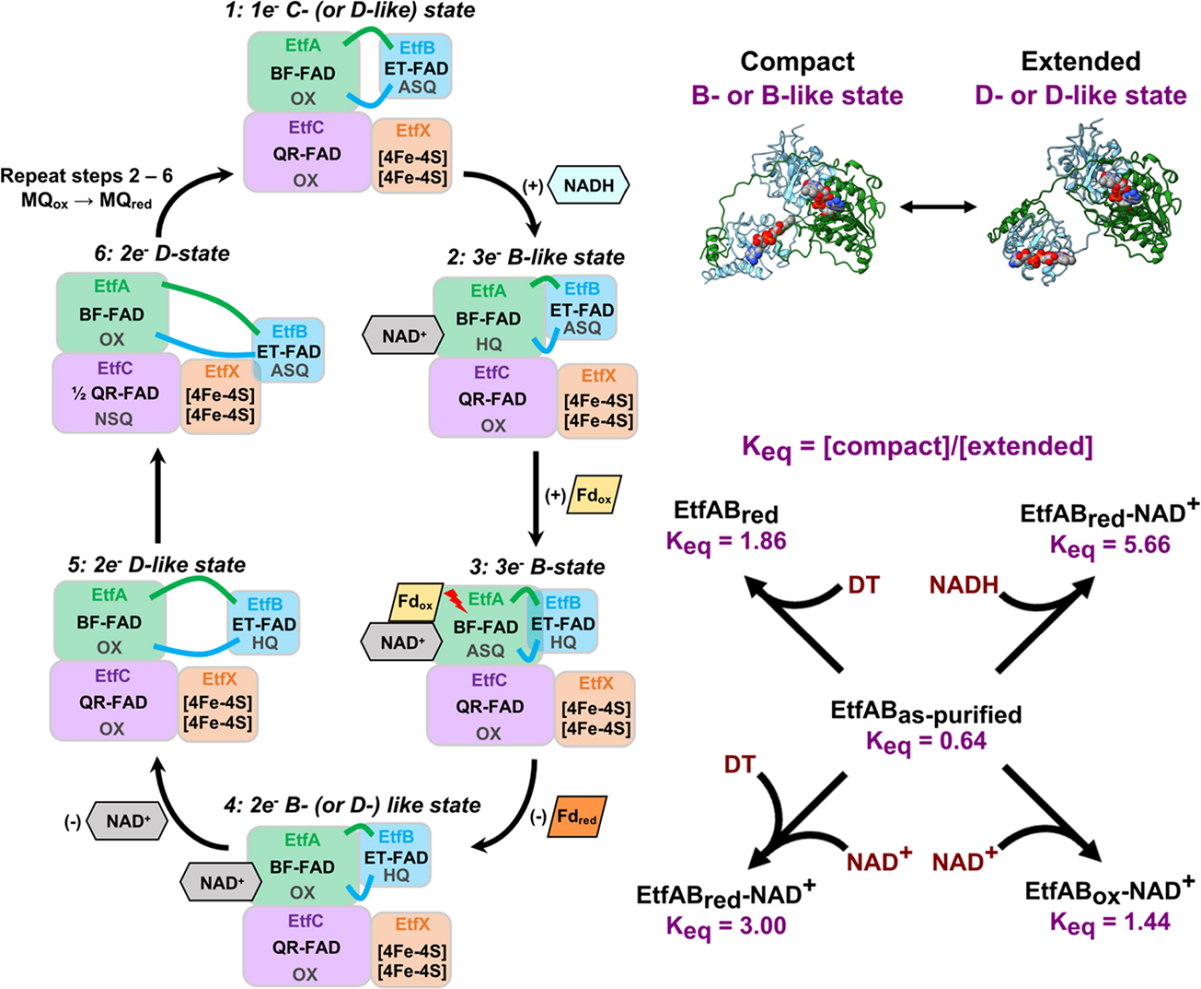
Correlating protein conformations with catalysis in EtfABCX. Correlation of EtfABCX’s structural states with the steps in its catalytic cycle (left). Upon flavin reduction and NAD(H) binding, EtfAB transitions from C- or D-like (C/D-like) states and compacts into B/B-like states. BF requires compaction by EtfAB in order for electronic coupling of BF- and ET-FAD. After BF, an extended conformation with respect to EtfAB’s core and shuttle domains is necessary for subsequent ET through the complex toward the MQ substrate. Compact and extended conformations of EtfAB (upper right)—represented here by B-like and D-like states—populate a two-state equilibrium that is influenced by solution, redox, and coenzyme factors. The *K*_eq_ values for each condition measured are shown (lower right), where larger values are indicative of equilibria more favoring compaction and vice versa.

We sought to place our results and those from other structural studies into the context of EtfABCX’s catalytic cycle (Figure 7). In the productive cycle, we start with the resting state where no NAD(H) is bound. The UV–visible spectra of the EtfAB(CX) resting state indicated that one of their flavins is in the FAD^·^**^–^** (ASQ) 1e^–^ reduced state (Figure S9).^13^ However, our resting state data from EtfABCX did not fully agree with the cryo-EM structure. This may be partly due to the cryo-EM associated selection of particles and picking a conformation that provides the highest resolution, aided by enforcing 2-fold symmetry.

To further isolate the observed differences between solution and static structures and their mechanistic implications, we applied a divide-and-conquer approach and characterized the conformational equilibrium for the EtfAB subcomplex. Our analysis of the resting state of EtfAB shows a C- and D-state mixture with 39% compact and 61% extended (Table 2 and Figure 7, Step 1). In later steps of the cycles (going clockwise in Figure 7 from the resting state) where electrons must be funneled to MQ, the ability for EtfAB to sample a D-like state in the context of EtfABCX is likely to be functionally important. In a B-like or even C-like state, the ability for ET-FAD to pass electrons to the [4Fe–4S] clusters of EtfX is prohibitive, based on the distance and intervening protein. By sampling a D-like state, these [4Fe–4S] clusters may be brought transiently closer for electron transfer, where even [4Fe–4S] cluster I may be accessible. This is intriguing in that, thus far, the need for [4Fe–4S] cluster I has not been explained.^10^ Perhaps, both [4Fe–4S] clusters I and II are used to shuttle electrons to the QR-FAD and finally to MQ.

The second step of a productive catalytic cycle (Figure 7, Step 2) is triggered by the binding and oxidation of NADH, the donation of electrons to BF-FAD. Following NADH oxidation, NAD^+^ is bound to EtfA, BF-FAD is in an HQ (2e^–^) state, and ET-FAD is in an ASQ state. However, the transfer of electrons between BF- and ET-FAD can occur only when EtfAB adopts a compacted conformation. In support of such a motion, we observed a shift in the conformational equilibrium of EtfAB in the presence of excess NADH, with 85% of the protein in solution adopting a B-like conformation where Domain II rotates and moves toward BF-FAD (Figures 3A and 5). Conformational changes of this kind have been demonstrated crystallographically in both nonbifurcating^46^ and bifurcating ETFs^11^ and have been inferred in accounting for the uncrossing of the BF-FAD’s half-potentials seen in the course of reductive half-reaction studies with the related *Megasphaera elsdenii* and *P. aerophilum* EtfABs.^47^

Structurally probing the next sequential step in the catalytic cycle (Figure 7, Step 3) is complicated by the involvement of Fd because its interactions with EtfABCX are thought to be transient. In the case of the NAD(H) coenzyme, SEC-SAXS measurements were conducted with excess concentrations in the running buffers. Assembling sufficient Fd for analogous experiments was prohibitive. However, for BF to occur, BF-FAD and ET-FAD must be as close if not closer (B-state) than what we had observed upon the addition of NADH.

The D-like state observed in extended EtfAB conformations likely promotes the transfer of reducing equivalents between ET-FAD and [4Fe–4S] II clusters of EtfX in the superdimer and subsequently to QR-FAD in EtfC (Figure 7, Steps 4–6). The flexibility enabled by the loops tethering EtfAB’s core to the mobile Domain II and the elongated position as observed in the D-like state make ET to the [4Fe–4S] II cluster in EtfX possible, surpassing the 14 Å threshold needed for productive electronic coupling between cofactors. Moving progressively through the high-potential pathway, this domain movement would oxidize HQ ET-FAD to ASQ and reduce QR-FAD to neutral semiquinone (NSQ; 1e^–^) after ET mediated by [4Fe–4S] II. An identical D-like/D-state would be required to transfer the next reducing equivalent during the second step of the catalytic cycle (Figures 1B and 7). This would bring QR-FAD to the HQ state in preparation for the final step of EtfABCX’s catalysis: the two-electron reduction of MQ.

### NAD(H)-Binding and FAD Redox State Trigger Separate Conformational Changes to EtfAB(CX)

The structure of an *A. fermentans* Etf-NAD^+^ complex was solved using MX after cocrystallizing EtfAB and NAD^+^.^11^ This showed NAD^+^ bound through interactions with β-FAD (BF-FAD in *T. maritima* EtfAB) and suggests that induced displacement by NADH upon binding β-FAD may affect the conformation of the C-terminal arm and β-hairpin of subunit β (*T. maritima*’s EtfA), which may be necessary to rotate and move Domain II closer to the core domain for ET. Of the conditions we were able to probe experimentally, the addition of NAD^+^ to the resting state is not expected to be on the pathway in a productive catalytic cycle. Quite the opposite, the expectation is that the relaxed state should preferentially release NAD^+^ for NADH. Interestingly, we observed a large conformational change in EtfABCX relative to the resting state and further from that observed when we added NADH (Figure 6B). As discussed above, the spectroscopic evidence suggests that NADH is rapidly converted into NAD^+^, which means that despite having the same coenzyme bound, the system adopts a different conformation. The observation of the changes in the three solution conditions (resting, excess NADH, and excess NAD^+^) indicates that both coenzyme binding and the redox state of the flavins can affect conformation. Modeling the excess NAD^+^ condition indicates a larger fraction of EtfAB is in a D-like state relative to excess NADH (41% relative to 15%, respectively).

The titration of NADH with EtfAB has been used in the literature as a proxy for setting the redox state of the system.^13,14,47^ Using 0, 0.5, 1, or 10 equiv of NADH, the system has been characterized with SAXS in the 0e^–^, 1e^–^, 2e^–^, and excess e^–^-reduced states, respectively. In conjunction, the *A*_374_/*A*_454_ absorption ratio has been used to monitor FAD redox states, particularly by probing the amount of FAD in the ASQ state. On a global structural level, SAXS provides an excellent readout of volume, which is related to flexibility as the oligomeric structure remains consistent in all assays. Utilizing this paradigm, we monitored the *A*_374_/*A*_454_ ratio and volume of EtfAB in the presence of varying equiv of NADH (Figure 4). We see that the *A*_374_/*A*_454_ absorption ratio is generally correlated to volume. A peculiar and outlier feature of characterizations of EtfAB in a 4e^–^ reduced state is that the absorption ratio is closer to that under less reduced conditions. This has been attributed to EtfAB entering a mixture of lesser-reduced states. This is possible through intermolecular ET, such as where BF-FAD is HQ and ET-FAD is ASQ, leading to a comproportionation reaction between EtfAB molecules yielding one EtfAB with its ET-FAD in an HQ state (2e^–^ per enzyme; BF-FAD oxidized) and another with both FADs as HQ (4e^–^ per enzyme) (Figure S14).^48^ As such, redox transfer between separate EtfAB molecules has been reported^48^ and, especially relevant given the length scale of an SEC-SAXS experiment, proteins in solution are assumed to reach redox equilibrium. The correlation observed between *A*_374_/*A*_454_ and the volume is attributed to the general compaction of EtfAB with respect to its domains upon reduction with NADH.

To better solidify that NAD(H) binding and FAD redox state separately affect conformational states, we adjusted the redox state with DT and then mixed in NAD^+^. DT is capable of reducing FAD in other flavoprotein systems.^49−51^ As noted above, the addition of NAD^+^ to EtfAB generates more extended conformations relative to those when NADH is added. However, when NAD^+^ is added to DT-reduced EtfAB, the system adopts a mixture of conformations that approach those observed with NADH (Table 2 and Figure 3C). This latter state mimics both coenzyme binding and the redox state once NADH is bound and oxidized. While the structural effect is not total, with a *K*_eq_ of 3 versus 5.66 (Figure 7; DT/NAD^+^ and NAD(H) conditions, respectively), the movement toward that observed in excess NADH is highly supportive of separate coenzyme and redox structural effects.

## Conclusions

Electron bifurcation by EtfABCX is an example of an evolutionarily ancient and newly identified form of biological energy conservation that creates high-energy electrons by elevating one electron at the expense of a second. The availability of a high-resolution cryo-EM structure of *T. maritima* EtfABCX has provided foundational information detailing the organization of its flavin and iron–sulfur cluster cofactors, MQ substrates, and superdimer protein scaffold. Furthermore, the system’s catalysis is understood to involve interactions with soluble NADH and Fd, that either donate or accept electrons, respectively. However, the pathway for electron flow is not immediately apparent from the single available structure and it is apparent that conformational changes are required.

Here, we observe EtfABCX’s structural changes and identify their triggers using an SEC-coupled solution scattering approach that can be applied to oxygen- and redox-sensitive BF-enzymes, in general. We have shown that both NAD(H) binding and the various electronic states of the EtfAB subcomplex and the EtfABCX holoenzyme separately modify structure. We provide evidence of conformational dynamics involving domain motions that enable the productive electronic coupling of cofactors. We have shown that the conformational search necessary for NADH oxidation, the adoption of BF-associated states, and subsequent ET in EtfABCX is best described by a two-state equilibrium. The equilibrium is influenced by coenzyme binding and the redox state of the cofactors in the enzyme and by the fact that this conformational equilibrium includes newly identified bifurcation-like and efficient electron conducting-like states. The conformational states identified here can be correlated with steps in the catalytic cycle of this enzyme to give a structural basis for its BF and ET mechanisms.

## Supporting Information Available

Additional sample details, SAXS parameters, biophysical analyses, SAXS profiles, ultraviolet–visible spectra, and model fits (PDF)

## Accession Codes

*Thermotoga maritima* Fix/EtfABCX: EtfA Tm1530 WP_004081902 EtfB Tm1531 WP_004081904 EtfC Tm1532 WP_004081907 EtfX Tm1533 WP_004081910
