# Supplementary material for: Correlating Conformational Equilibria with Catalysis in the Electron Bifurcating EtfABCX of Thermotoga maritima
Source: Biochemistry. 2023 Nov 28;63(1):128–40. doi: 10.1021/acs.biochem.3c00472 (PMC10765413; doi:10.1021/acs.biochem.3c00472)
Supplement: Supplementary file 1 — bi3c00472_si_001.pdf [file bi3c00472_si_001.pdf]

Supporting Information

Correlating conformational equilibria with catalysis in the electron bifurcating EtfABCX of *Thermotoga maritima*

Daniel T. Murray[1], Xiaoxuan Ge[2], Gerrit J. Schut[2], Daniel J. Rosenberg[1,3], Michal Hammel[1], Jan C. Bierma[1], Russ Hille[4], Michael W. W. Adams[2], and Greg L. Hura[1,5,*]

[1]Molecular Biophysics and Integrated Bioimaging Division, Lawrence Berkeley National Laboratory, Berkeley, CA, USA

[2]Department of Biochemistry and Molecular Biology, University of Georgia, Athens, GA, USA

[3]Linac Coherent Light Source, SLAC National Accelerator Laboratory, Menlo Park, CA, USA

[4]Department of Biochemistry, University of California, Riverside, Riverside, CA, USA

[5]Chemistry and Biochemistry Department, University of California, Santa Cruz, Santa Cruz, CA, USA

***Correspondence:** Greg L. Hura: glhura@lbl.gov

Supplementary Table S1 – S2

Supplementary Figures S1 – S14

Supplementary Table S1. Sample details for anaerobic SEC-MALS-SAXS experiments. [a]Standard buffer is 20 mM Tris-HCL, pH 8.0, 200 mM NaCl. [b]20 Eq additive ensured each EtfAB subcomplex was exposed to 10 Eq additive.

Protein	Oligomer / subunit composition	Condition / Additive	[Protein] (mg/mL)	Buffer / % DDM	Additive molar equivalents (Eq)	Additive in running buffer or preincubated	Injection volume (μL)
EtfABCX	Superdimer / $(ABCX)_2$	No additive	6	Standard[a] / 0.01	0	Running buffer	55
		NADH			10[b]		
		NAD^+					
		DT	7.5				
EtfAB	Heterodimer subcomplex / AB	No additive	4	Standard / 0	0	Running buffer	95
		NADH			8.5		
		NAD^+					
		DT	4.75		7.35		
		DT and NAD					
		Air-oxidized	4.86		0	Preincubated	
		$1e^-$ (NADH)			0.5		
		$2e^-$ (NADH)			1		
		$4e^-$ (NADH)			2		

Supplementary Table S2. Primary SAXS analysis of EtfAB samples containing varying NADH equivalents along with absorption values for FAD. Molecular volume parameters are included for those mentioned in the main text.

Sample	Condition	Guinier R_g, Å	IFT R_g, Å[b]	D_{max}, Å	Volume, Å^3	A_{374}	A_{454}
EtfAB	Air-oxidized	27.1 ± 0.1	27.5 ± 0.1	91	128,524	91.4	104.8
	$1e^-$ (NADH)	26.6 ± 0.2	27.0 ± 0.1	79	123,523	104.2	105.6
	$2e^-$ (NADH)	27.9 ± 0.2	28.2 ± 0.1	98	113,985	87.7	68.5
	$4e^-$ (NADH)	28.9 ± 0.1	29.5 ± 0.1	103	119,953	88.5	68.3
	Excess e^- (NADH)	See "Table 1" in the text.			111,841	97.0	45.9
	As-purified ("No additive")				117,658	126.6	130.3

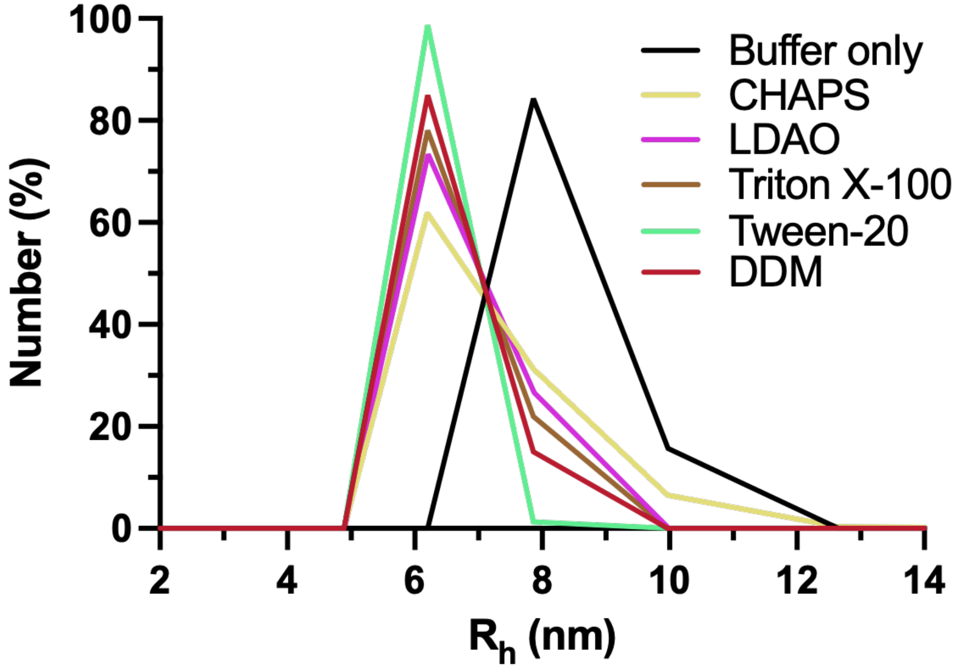

Figure S1. Detergents shift EtfABCX's oligomeric equilibrium towards the superdimer as shown by dynamic light scattering. Upon the addition of detergent, the hydrodynamic radius (R_h) distribution of EtfABCX shifts from ~8 nm to ~6 nm, representing a shift from supertetramer to superdimer states. Each detergent condition was measured in duplicate and as concentration series. Representative R_h distributions for each detergent are shown for clarity.

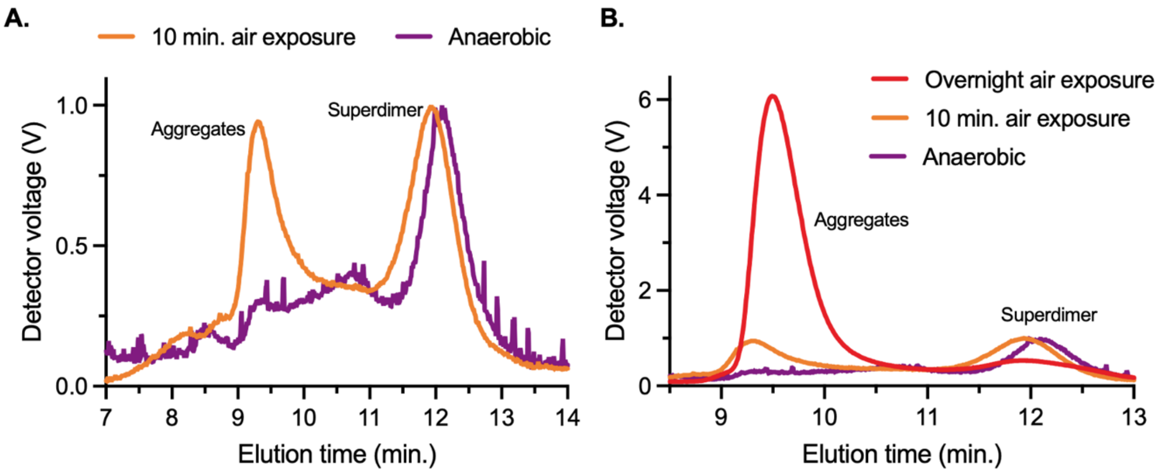

Figure S2. EtfABCX exhibits time-dependent sensitivity to oxygen-induced aggregation as analyzed with SEC-MALS. A. SEC-MALS traces of EtfABCX specimen as prepared anaerobically or after 10 minutes of air exposure prior to sample injection. **B.** SEC-MALS traces as shown in **A** with the addition of an overnight air exposure sample.

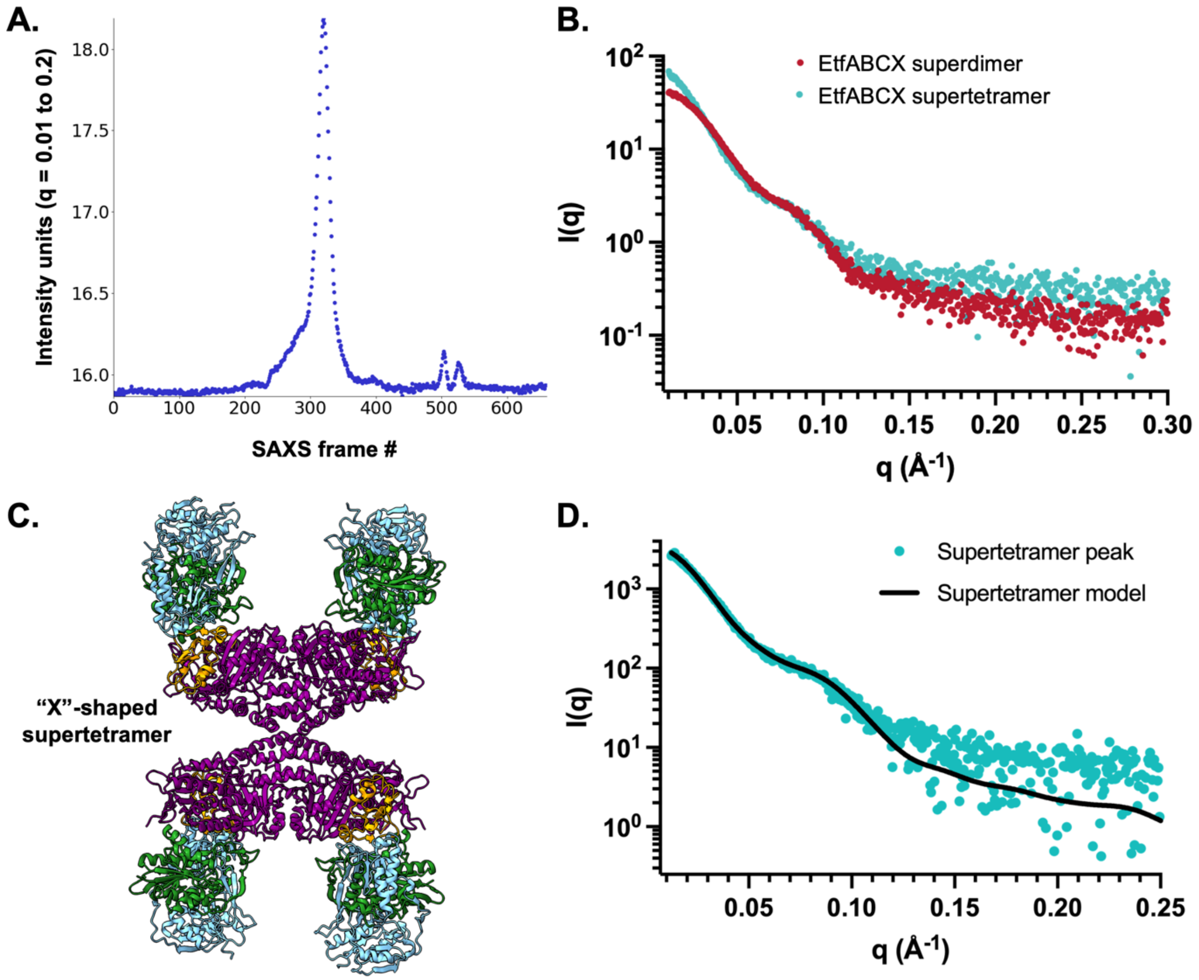

Figure S3. Anaerobic SEC-SAXS using 0.01% (w/v) DDM-containing running buffer isolates the SAXS signal of EtfABCX superdimer. A. SEC-SAXS trace of a representative EtfABCX specimen (no additives) showing a clearly defined peak for its superdimer and a minimal supertetramer shoulder. **B.** Merged SAXS profiles from both superdimer peak and supertetramer shoulder showing distinctly isolated scattering curves. Superdimer and supertetramer scattering profiles yielded SAXS MW values of 244 and 474 kDa, respectively (257 kDa expected for superdimer). **C.** An "X"-shaped EtfABCX supertetramer. **D.** The calculated profile for an EtfABCX supertetramer overlaid with the experimental scattering obtained from the supertetramer shoulder apparent during SEC-SAXS of NADH-reduced sample. The agreement at low to mid q supports the oligomeric state of the supertetramer.

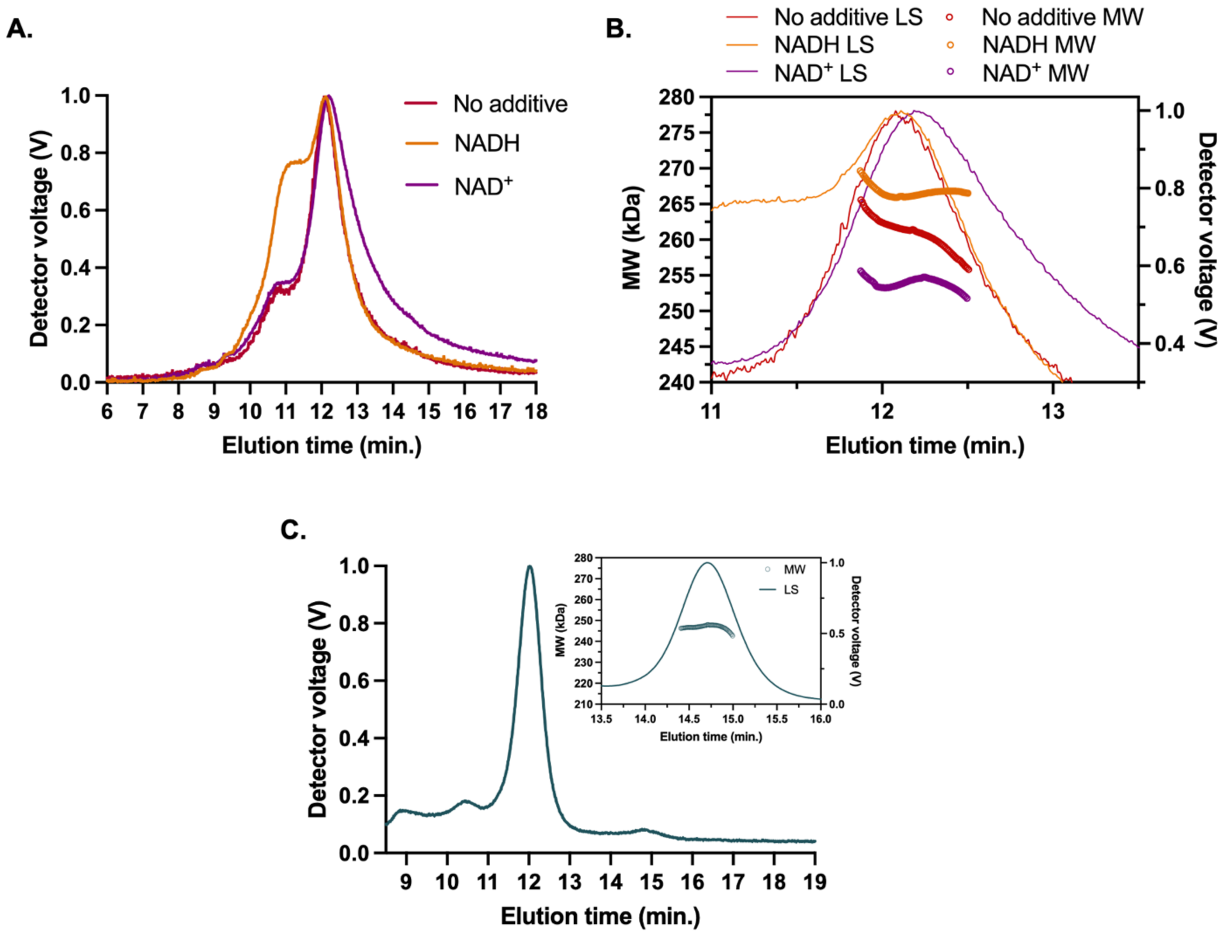

Figure S4. In-line MALS collected during anaerobic SEC-MALS-SAXS show EtfABCX specimen peaks are characterized by species of the expected MW. A. Normalized MALS traces for no additive, NADH, and NAD^+ conditions for EtfABCX overlaid as collected during SEC-MALS-SAXS. **B.** MW analysis on the MALS data in (**A**) shows EtfABCX superdimer peaks contain species within +/- 5% of the expected MW for a 9x histidine-tagged superdimer (257 kDa; LS = light scattering signal). **C**. SEC-MALS of EtfABCX with DT reduction shows steady MW values across its peak (inset) and absence of supertetramer formation. DT-containing datasets are separated from preceding panels for clarity.

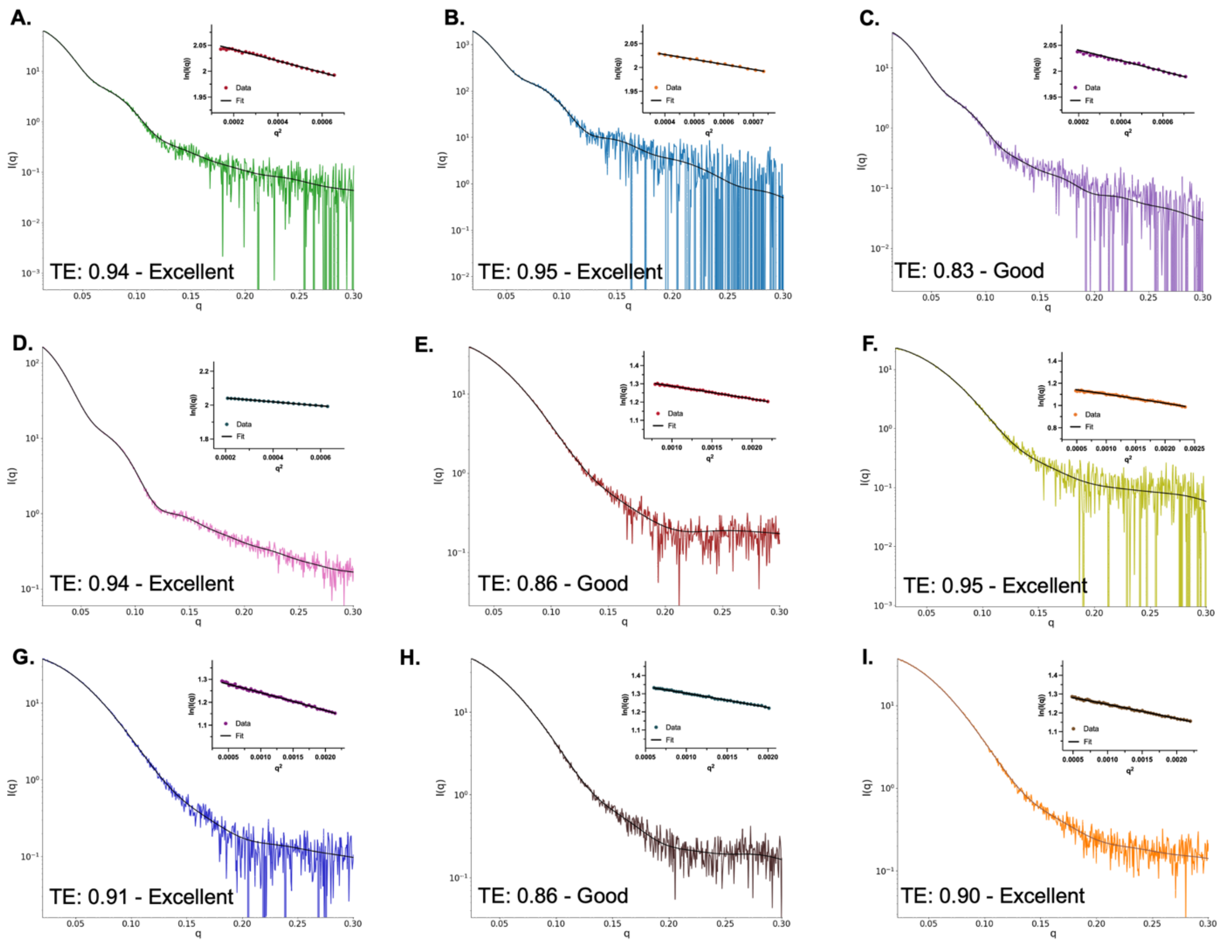

Figure S5. SAXS profiles along with their respective Guinier and P(r) fits for EtfABCX and EtfAB specimens. Colored lines represent experimental data. Black lines represent calculated fits for P(r) functions. Total estimates (TE) from GNOM are provided. Insets: Guinier fits. Error bars represent the standard deviation of I(q). **A.** EtfABCX with no additives. **B.** EtfABCX with NADH. **C.** EtfABCX with NAD^+. **D.** EtfABCX with DT. **E.** EtfAB with no additives. **F.** EtfAB with NADH. **G.** EtfAB with NAD^+. **H.** EtfAB with DT. **I.** EtfAB with DT and NAD.

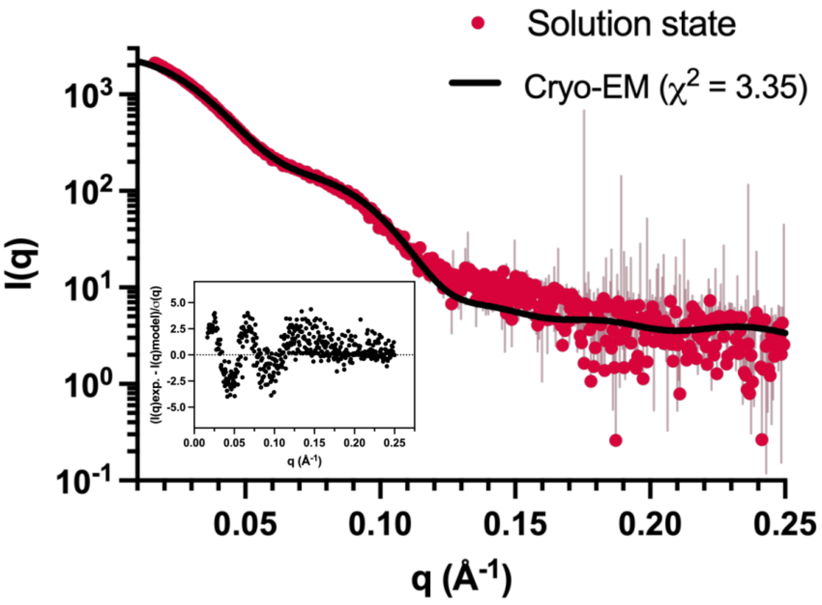

Figure S6. Comparison of EtfABCX's SAXS profile with the computed profile for its cryo-EM structure. The black line represents the computed profile for the cryo-EM structure (PDB ID: 7KOE). Inset: residuals. Error bars represent the standard deviation of I(q).

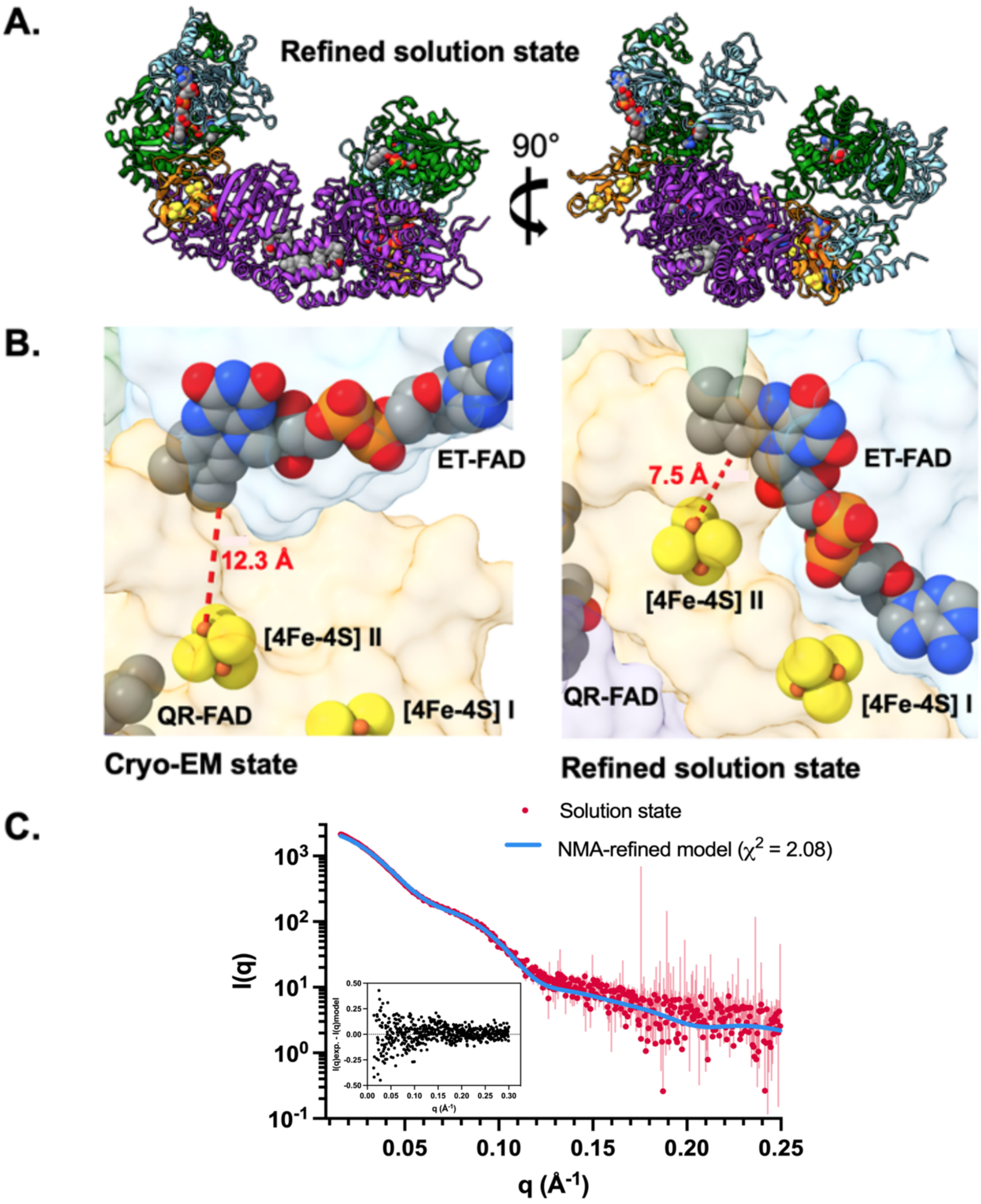

Figure S7. EtfABCX's supermonomers exhibit varying spatial correlations, in turn affecting cofactor distancing. A. Flexibly refined model of EtfABCX possessing a "twisted" configuration with respect to supermonomer conformation. **B**. End-to-end distances between ET-FAD and [4Fe-4S] II cofactors in cryo-EM (left) and refined model (right). ET-FAD and [4Fe-4S] II distances in the refined model resemble those in D- or D-like states. **C**. Fit of the NMA-refined model's calculated profile to the SAXS data. Inset: residuals. Error bars represent the standard deviation of I(q).

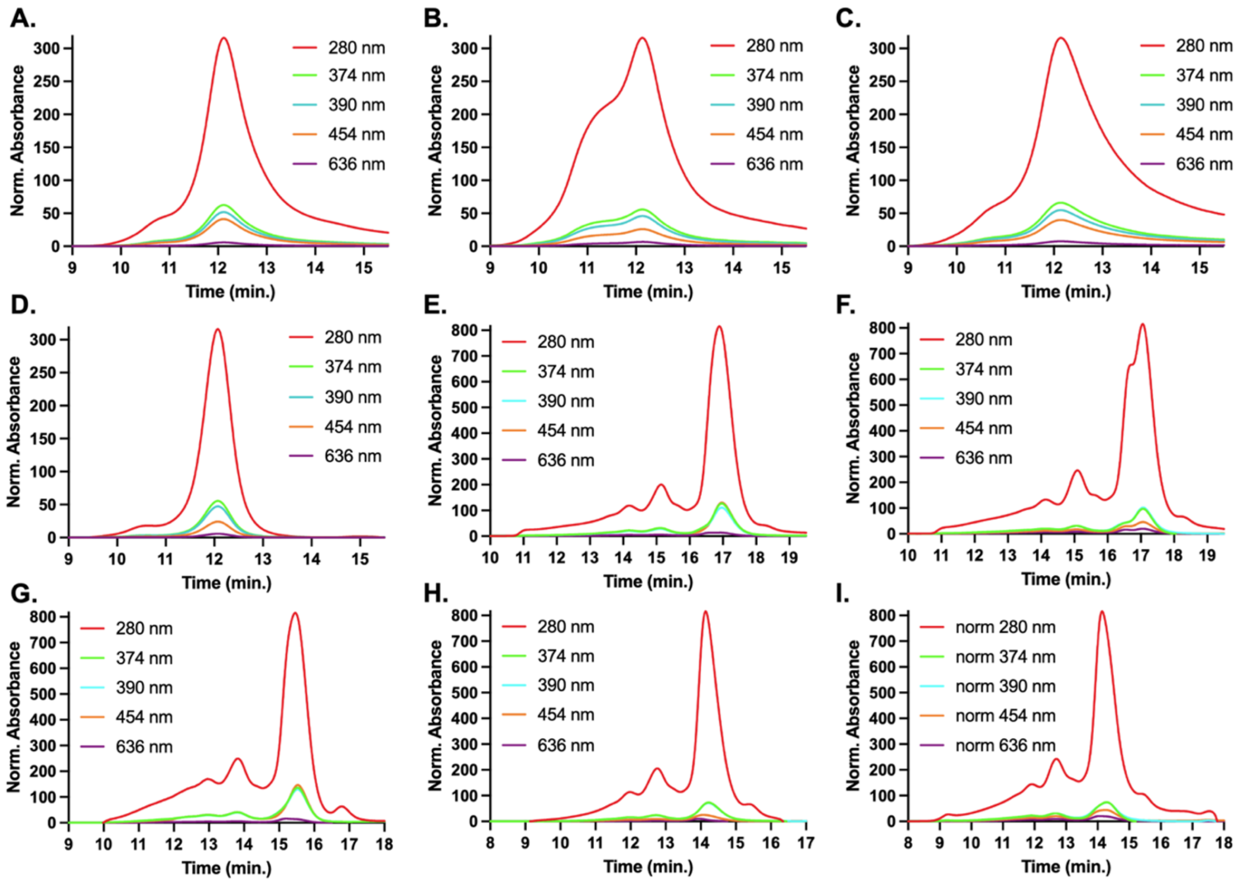

Figure S8. In-line UV-visible spectra collected during anaerobic SEC-MALS-SAXS. The absorbances at 280, 374, 390, 454, and 636 nm were recorded during measurements of EtfABCX in no additive (**A**), NADH (**B**), NAD^+ (**C**), and DT (**D**) conditions, allowing for the monitoring of redox state and coenzyme binding. Similarly, spectra were recorded during measurements of EtfAB in no additive (**E**), NADH (**F**), NAD^+ (**G**), DT (**H**), and DT/NAD^+ (**I**) conditions. Spectra were normalized to the A_{280} maxima of the no additive conditions' EtfABCX superdimer or EtfAB heterodimer peaks, respectively.

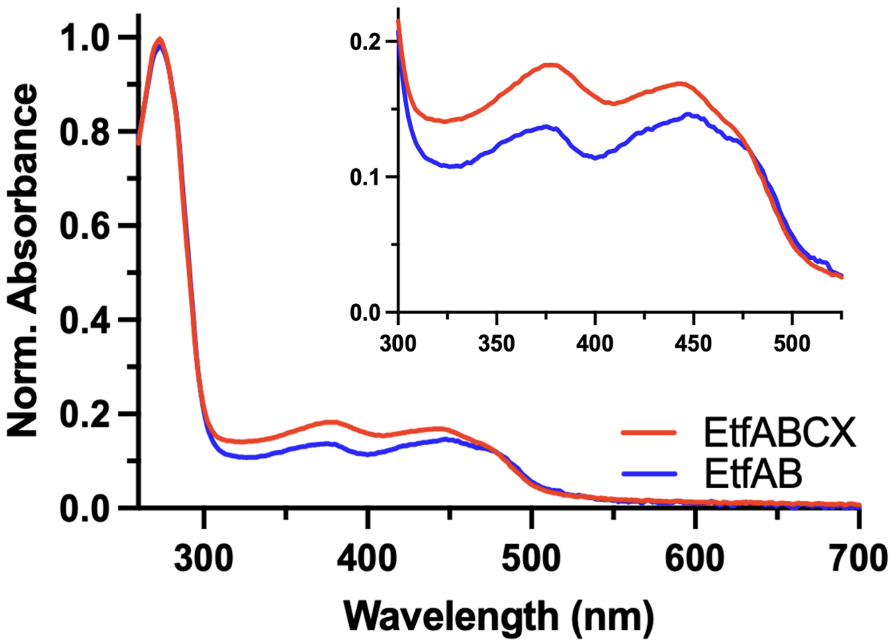

Figure S9. UV-visible absorbance spectra of as-purified "no additive" *T. maritima* EtfABCX and EtfAB as expressed and purified from *E. coli*. Normalized absorbance peaks centered around 374 and 454 nm correspond to cofactor occupancy.

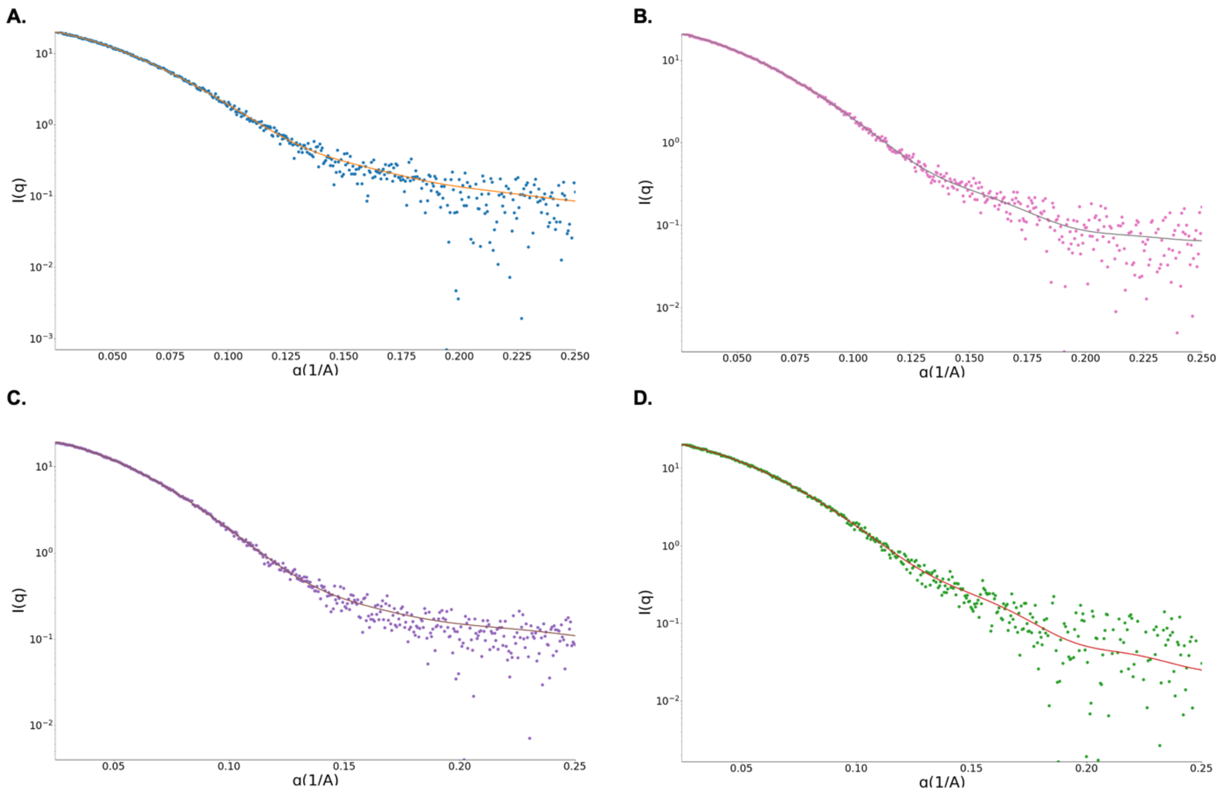

Figure S10. SAXS profiles and P(r) fits for EtfAB specimens preincubated with the NADH concentration series. A. Air-oxidized. **B.** 0.5 Eq NADH / 1e$^-$. **C.** 1 Eq NADH / 2 e$^-$. **D.** 2 Eq NADH / 4 e$^-$.

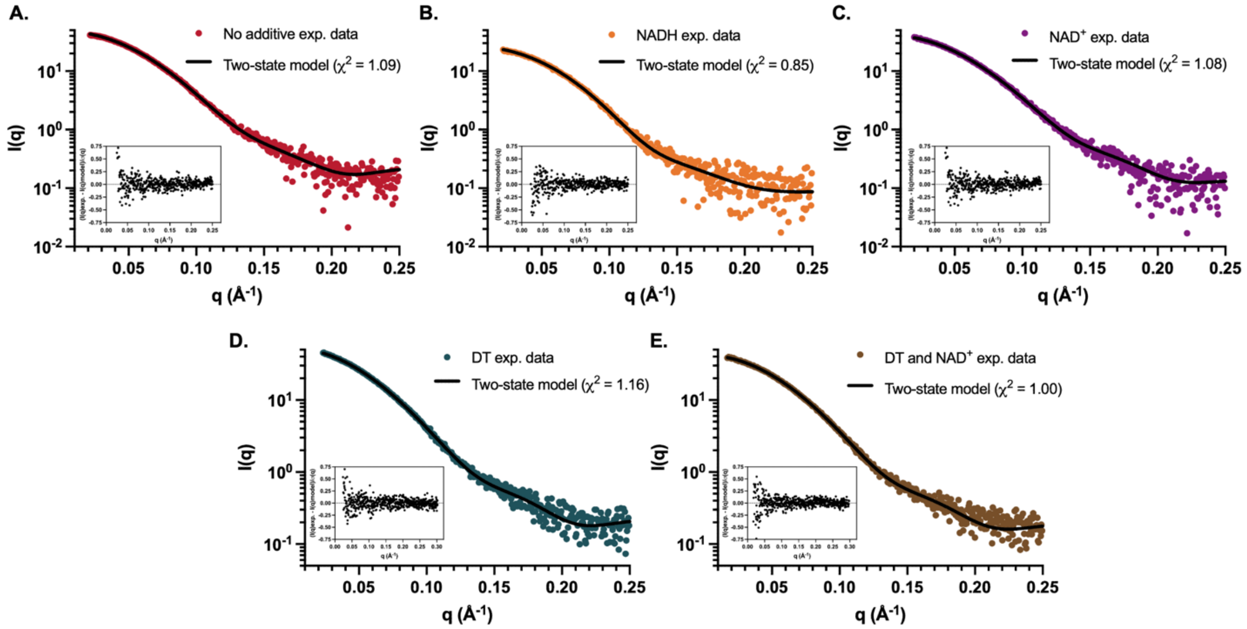

Figure S11. Fits of EtfAB's two-state models to their corresponding experimental data. The overlay of EtfAB's SAXS profiles with that of the fits (black lines) from two-state models as generated with BilboMD. Insets: residuals. **A.** EtfAB in the no additive condition. **B.** Same as in **A** but for EtfAB's NADH condition. **C.** Fit of the two-state model for EtfAB's NAD^+ condition. **D.** Fit of the two-state model for EtfAB's DT condition. **E.** Fit of the two-state model for EtfAB's DT and NAD^+ condition.

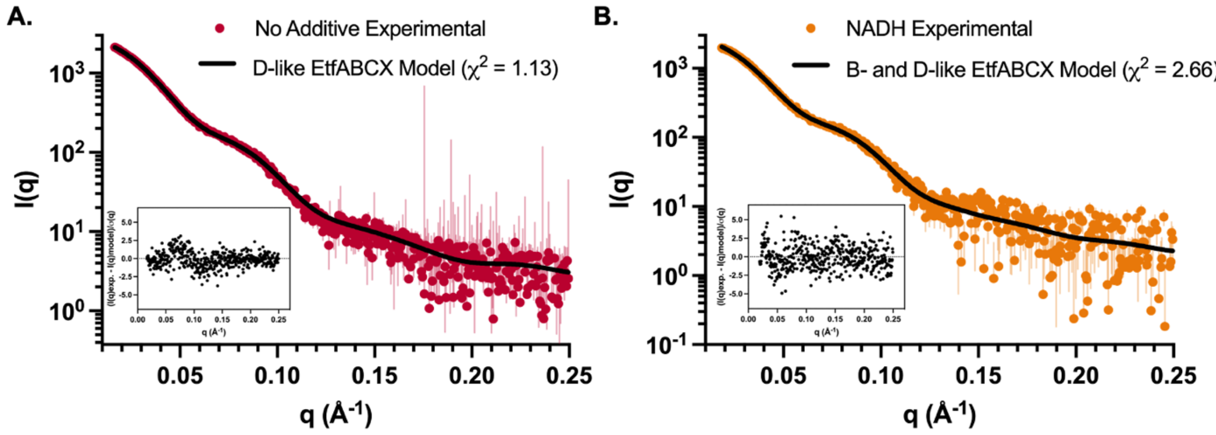

Figure S12. The fit of EtfABCX models to their corresponding experimental data. Insets: residuals. **A.** No additive condition's data with fit of EtfABCX possessing EtfAB modules in D-like conformations. **B.** NADH condition's data with fit of EtfABCX possessing EtfAB modules in B- and D-like conformations. The models and their respective fits shown here were the highest scored from among the possibilities tested. Error bars represent the standard deviation of I(q).

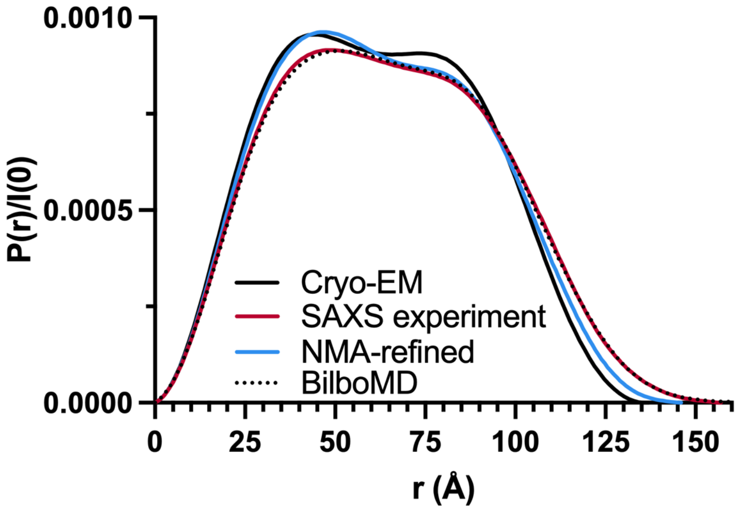

Figure S13. Comparison of P(r) functions for EtfABCX's structural models. Overlaid are the P(r) functions calculated from EtfABCX's cryo-EM state, primary analysis of the experimental "no additive" SAXS data, the NMA-refined structure based on the "no additive" SAXS data, and the best-fitting BilboMD-derived model for the "no additive" SAXS data with EtfABs in D-like states.

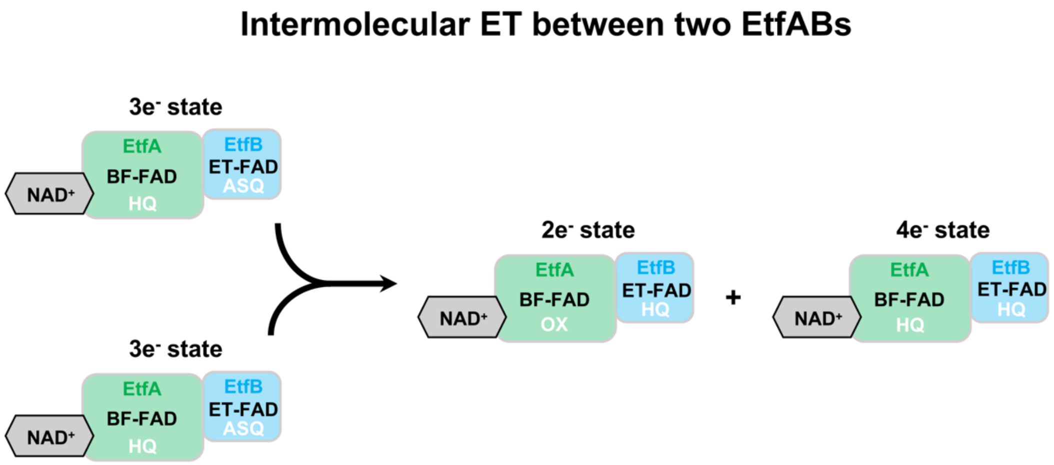

Figure S14. Potential for intermolecular electron transfer between EtfAB . Two partially-reduced EtfABs may perform intermolecular ET to yield partially or fully reduced forms of the enzyme. The potential for intermolecular ET increases with incubation time.
